# Not that clean: Aquaculture‐mediated translocation of cleaner fish has led to hybridization on the northern edge of the species' range

**DOI:** 10.1111/eva.13220

**Published:** 2021-03-29

**Authors:** Ellika Faust, Eeva Jansson, Carl André, Kim Tallaksen Halvorsen, Geir Dahle, Halvor Knutsen, María Quintela, Kevin A. Glover

**Affiliations:** ^1^ Department of Marine Sciences – Tjärnö University of Gothenburg Strömstad Sweden; ^2^ Institute of Marine Research Bergen Norway; ^3^ Austevoll Research Station Institute of Marine Research Storebø Norway; ^4^ Institute of Marine Research His Norway; ^5^ Centre of Coastal Research University of Agder Kristiansand Norway; ^6^ Institute of Biology University of Bergen Bergen Norway

**Keywords:** aquaculture, genetic hybridization, human‐mediated gene flow, Labridae, parasites, single nucleotide polymorphism

## Abstract

Translocation and introduction of non‐native organisms can have major impacts on local populations and ecosystems. Nevertheless, translocations are common practices in agri‐ and aquaculture. Each year, millions of wild‐caught wrasses are transported large distances to be used as cleaner fish for parasite control in marine salmon farms. Recently, it was documented that translocated cleaner fish are able to escape and reproduce with local wild populations. This is especially a challenge in Norway, which is the world's largest salmon producer. Here, a panel of 84 informative SNPs was developed to identify the presence of nonlocal corkwing wrasse (*Symphodus melops*) escapees and admixed individuals in wild populations in western Norway. Applying this panel to ~2000 individuals, escapees and hybrids were found to constitute up to 20% of the local population at the northern edge of the species’ distribution. The introduction of southern genetic material at the northern edge of the species distribution range has altered the local genetic composition and could obstruct local adaptation and further range expansion. Surprisingly, in other parts of the species distribution where salmon farming is also common, few escapees and hybrids were found. Why hybridization seems to be common only in the far north is discussed in the context of demographic and transport history. However, the current lack of reporting of escapes makes it difficult to evaluate possible causes for why some aquaculture‐dense areas have more escapees and hybrids than others. The results obtained in this study, and the observed high genomic divergence between the main export and import regions, puts the sustainability of mass translocation of nonlocal wild wrasse into question and suggests that the current management regime needs re‐evaluation.

## 1 INTRODUCTION

Moving organisms outside their natural boundaries can cause diverse effects on the ecosystems (Atalah & Sanchez‐Jerez, 2020). Introductions can affect some species through ecological competition, either by becoming their prey or predator, or by competing for resources (Evangelista et al., 2019). Introduced individuals can also carry pathogens, that being unknown to the local population, can spread quickly into a novel environment, which has not been able to develop any form of resistance (Tepolt et al., 2020). Furthermore, if the introduced populations are genetically distinct from the local ones, hybridization and admixture can lead to altered population structure (Glover et al., 2012), lower effective population size and reduced fitness through outbreeding depression (Blakeslee et al., 2020; Glover et al., 2017; Laikre et al., 2010). Donor populations and ecosystems can also be negatively affected if harvest leads to disruption in species interactions and ecosystem function (Halvorsen, Larsen, et al., 2017). Adverse genetic effects, such as loss of diversity due to dwindling population size or selective harvesting, can also be experienced (Allendorf et al., 2008). However, and despite their known adverse effects, the introduction of species into new areas and translocation of individuals from foreign populations are still common practice in aquaculture and fisheries management. Translocations aim to increase catches, mitigate loss of wild stocks and restore or even create new fisheries (Laikre et al., 2010). Likewise, many species are harvested in large numbers in the wild to provide food or other services to cultured species such as cleaner fish to delouse salmonids.

The use of cleaner fish for sea lice control in commercial aquaculture was first implemented in the late 1980s (Bjordal, 1988) and increased dramatically from 2008 onwards as a result of sea lice developing resistance to widely used pharmaceutical treatments (Besnier et al., 2014; Fjørtoft et al., 2020; Kaur et al., 2017). In Norway alone, the number of cleaner fish used increased from 1.7 million in 2008 to 60 million in 2019 (Norwegian Directorate of Fisheries, 2019). Outside Norway, the use of cleaner fish in parasite control is still relatively limited but set to increase (VKM et al., 2019). Some countries, such as UK and Ireland, apply a similar system to Norway by deploying a mixture of farmed and wild‐caught cleaner fish (Bolton‐Warberg, 2018; Riley et al., 2017) while others, for example Canada, do not allow the use of wild‐caught cleaner fish in open marine aquaculture (Boyce et al., 2018). The possibility to use cleaner fish for parasite control in aquaculture is currently being investigated in other salmon‐producing countries as well (Sanchez et al., 2018).

At present, there are five different species used as cleaner fish in Norwegian aquaculture: lumpfish (*Cyclopterus lumpus*), ballan wrasse (*Labrus bergylta*), goldsinny wrasse (*Ctenolabrus rupestris*), corkwing wrasse *(Symphodus melops*) and rock cook (*Centrolabrus exoletus*), the latter in lower numbers. Lumpfish, whose potential use as a cleaner fish was discovered in 2014, has since become the most commonly used cleaner fish (Imsland et al., 2014). The majority of lumpfish are farmed while almost all wrasses are caught wild and transported to aquaculture facilities. Currently, ballan wrasse is the only commercially reared wrasse species, albeit still at a relatively small scale (Norwegian Directorate of Fisheries, 2019). Goldsinny and corkwing wrasse are, by far, the most commonly used wild‐caught cleaner fish. In 2019, 7.9 million goldsinny and 7.3 million corkwing wrasse, all captured in the wild, were deployed as cleaner fish in Norwegian aquaculture.

Although often considered as an environmental friendly form of parasite control (Liu & Bjelland, 2014), the increasing fishing pressure and large‐scale translocation of cleaner fish raise concerns about potential overfishing and human‐mediated gene flow from translocated individuals to wild populations. Animal welfare during transportation and in sea cages is also a matter of concern (Geitung et al., 2020). An estimated 1 million wrasse are harvested in south‐western England and transported to Scottish salmon farms (Davies & West, 2017; Riley et al., 2017). In Norway, millions of wrasses are utilized as cleaner fish and translocated hundreds of kilometres to be used in salmon farms (VKM et al., 2019). There are many examples of salmonids escaping open‐pen aquaculture and hybridizing with local populations, leading to genetic swamping and reduced fitness (Bolstad et al., 2017; Glover et al., 2017). Recently, several studies have collectively demonstrated that also wrasses are able to escape from salmon farms and potentially hybridize and admix with local populations (Blanco Gonzalez et al., 2019; Faust et al., 2018; Jansson et al., 2017). However, the geographical extent, magnitude of genetic mixing and the ecological consequences are largely unknown. In contrast to regulations for salmonid farming, there are currently no requirements for preventing escape of cleaner fish from sea cages, nor reporting escapes when they occur.

Wrasses (*Labridae*) are a large and diverse family of marine fish with over 600 described species worldwide. Many of these species show natural cleaning behaviour, that is they feed on ectoparasites from other fish species’ skin. The wrasse species utilized as cleaner fish on Norwegian fish farms inhabit shallow rocky areas along the coast from the Mediterranean Sea in the south, to the Norwegian coast in the north. In recent years, their abundance has shifted northwards and diminished in the south, which has been suggested to be due to increased seawater temperatures (Knutsen et al., 2013). These species differ in their ecology and life history characteristics in several ways, but they are all believed to be territorial and nonmigratory, thus almost exclusively dependent on the planktonic early life stages for dispersal (Darwall et al., 1992; Halvorsen et al., 2020; Skiftesvik et al., 2014). Depending on species and the set geographic scope, previous studies of wrasses have shown varying degree of genetic population sub‐structuring (see D'Arcy et al., 2013; Jansson et al., 2017; Knutsen et al., 2013; Robalo et al., 2012; Seljestad et al., 2020). One striking feature is, however, the detected genetic break for corkwing, which is located at the south‐western tip of Norway around sandy beaches in Jæren and Lista (Blanco González et al., 2016). The break only spans <60 km and has been suggested to be a result of postglacial recolonization and founder events separating the populations for more than ~10 kya (Mattingsdal et al., 2020).

Corkwing wrasse is a nest‐building species that spawns benthic eggs, which are dependent on paternal care until hatching. Nesting males are brightly coloured and significantly larger than females or sneaker males, which mimic the females’ brown colour and smaller body size (Halvorsen et al., 2016). Currently, nesting males are disproportionately targeted by Norwegian fisheries, which are regulated by a minimum size limit (Halvorsen, Sørdalen, et al., 2017). However, size, maturity and proportion of nesting males to sneaker males do not seem to be consistent across populations. Recent studies suggest that populations south of the genetic break in south‐western Norway are growing faster, maturing earlier, having a shorter life span and a lower proportion of sneaker males to nesting males (Halvorsen et al., 2016).

The strong genetic differentiation found between corkwing populations located on the south vs. the west coast of Norway has allowed for the development of genomic tools to identify escapees as well as first‐ and second‐generation hybrids between southern individuals and local populations (Faust et al., 2018). Faust and colleagues showed in their study (2018) that translocated corkwing wrasse can escape and hybridize with local populations at the northern edge of the species current distribution limit in Flatanger, Norway. Of the 40 corkwing wrasse they collected, two were identified as southern escapees and 13 as potential first‐ or second‐generation hybrids. However, that proof‐of‐concept study was geographically limited, and only based on a low number of individuals. Therefore, more extensive sampling is needed in order to quantify the extent and magnitude of escapees and hybrids of wrasse from southern regions. In the present study, we addressed this by first developing an informative panel of genome‐wide SNPs, and then using this panel to analyse ~2000 corkwing wrasse collected from aquaculture‐dense regions in western Norway and potential source populations in Skagerrak‐Kattegat.

## 2 MATERIALS AND METHODS

### 2.1 SNP selection and bioinformatics

In order to find discriminant and divergent SNPs for the identification of nonlocal corkwing wrasse, we used 2b‐RAD sequence‐data from western Norway (import region) and Skagerrak‐Kattegat (export region). Western sample sequences were taken from Faust et al. (2018) and contained 40 individuals from Austevoll, the only region where the authors did not detect any escapees or potential hybrids. As a reference for the exported fish, we used 120 individuals from three locations in the Skagerrak‐Kattegat (Risør, Sandefjord and Kungsbacka). All raw sequences are available on NCBIs Sequence Read Archive (BioProject PRJNA702627). The unpublished sequences were sampled and processed in the same way as the ones from Austevoll using a modified version of 2b‐RAD (Wang et al., 2012) full procedure (Faust et al., 2018). All sequences were mapped using bowtie2 (Langmead & Salzberg, 2012) to the published *Symphodus melops* genome (Mattingsdal, 2017). Variant calling was done following the GATK pipeline (McKenna et al., 2010) using UnifiedGenotyper after realigning sequences around indels and recalibrating base quality (BQSR). Variant score quality was recalibrated (VQSR) using site identity across technical replicates as a training set. To ensure high confidence in genotypes and SNPs, we used vcftools (Danecek et al., 2011) filtering on quality by depth (QD < 2.0), strand bias (FS > 60, SOR > 2) and mapping quality (MQ < 40). Sites with more than 10% missing data and with a fraction of heterozygotes above 0.5 (possible lumped paralogs) were removed, leaving a total of 10 747 putative SNPs.

To select the most divergent SNPs between western and Skagerrak‐Kattegat individuals, we conducted pairwise comparisons between Austevoll (western Norway) and each of the three locations in Skagerrak‐Kattegat. A total of 387 SNPs, distributed over 270 contigs, were identified among the 500 highest *F*_ST_ values in all three pairwise comparisons. Reading and converting between file formats was done using VcfR radiator (Knaus & Grünwald, 2016, 2017) and Radiator (Gosselin, 2019), and the package diveRsity (Keenan et al., 2013) was used to calculate pairwise *F*_ST_.

SNPs displaying FST values >0.4 (183 SNPs total) were used for SNP locus primer design and resulted in four assays with a total of 106 SNPs. Primer design, amplification and genotype calling were based on the Agena MassARRAY iPLEX Platform, as described by Gabriel et al. (2009). Selected 106 SNP loci were analysed in four assay groups (Table S1). Accuracy, efficiency and power of the four assays to correctly identify escaping individuals from the two populations and their potential offspring were estimated using the R package HYBRIDDETECTIVE (Wringe et al., 2017a). Genotype frequencies from the reference samples in Austevoll and Risør with 40 individuals each were used to simulate three replicates of three independent data sets with pure parents (Pure1 and Pure2), first‐ and second‐generation hybrids (F1 and F2), and backcrosses between F1 and pure parents (BC1 and BC2). The simulated data sets contained 288 individuals and were analysed using the R package parallelnewhybrid (Wringe et al., 2017b) and NEWHYBRIDS v. 1.1 (Anderson & Thompson, 2002), which estimates the posterior probability of each individual to belong to one of the six hybrid classes. The analysis was done using default priors and genotype proportions, with a burn‐in period of 50,000 iteration and 300,000 MCMC sweeps. In case of nonconvergent MCMC chains, simulations were re‐analysed. Power was estimated as the product of efficiency (correctly assigned individuals over the known individuals per class) and accuracy (correctly assigned individuals over individuals assigned to that class) as described in Wringe et al. (2017a). Simulations demonstrated a high efficiency (>94%), accuracy (>98%) and power (>94) to detect individuals from all of the six hybrid classes (Figure S1).

### 2.2 Data collection and processing

#### 2.2.1 Sampling

**FIGURE 1:**
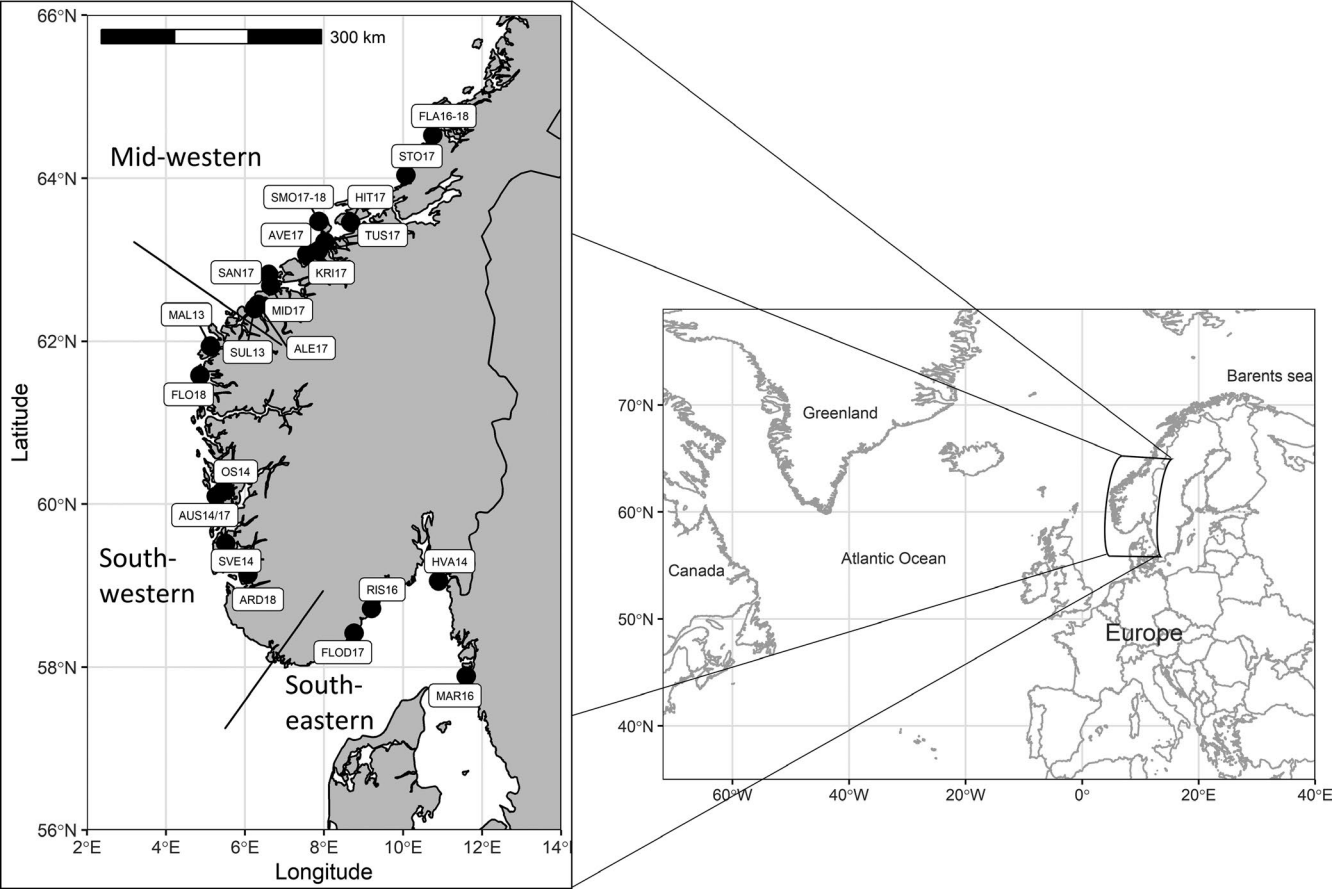
Corkwing wrasse sampling locations with respective abbreviations. Solid lines in the map indicate borders between regions. For details see Table 1

In total, 1954 corkwing wrasse were collected from 22 locations in western and mid‐Norway, which represents the primary region where cleaner fish originating from southern Norway and Sweden are translocated to delouse salmon on commercial farms (Table 1; Figure 1). As the aim was to cover a wide area and as many locations as possible, an opportunistic sampling scheme was introduced leading to very uneven sample sizes per location (range 1–365) and a time span of six years (from 2013 to 2018). Collection emphasis was focussed in mid‐Norway (counties of Trøndelag and Møre og Romsdal), which is the primary recipient area of translocated corkwing wrasses, and where the hybridization between local and translocated fish had already been reported (Faust et al., 2018). Five hundred fish were collected in three consecutive years (2016–2018) in Flatanger (FLA16‐18 in Figure 1), which roughly represents the species’ current northernmost distribution limit. Part of the 105 fish collected in 2016 (*N* = 40) were already used in Faust et al. (2018), whereas additional samples from 2017 (*N* = 365) and 2018 (*N* = 30) were collected for the current study. Smøla is an island municipality ~200 kilometres south from Flatanger with a high density of fish farms. In 2017–2018, 271 fish were collected there (SMO 17‐18 in Figure 1) to increase the sampling effort in mid‐western Norway. Additional 126 corkwing wrasses from 8 locations from mid‐Norway were obtained as by‐catch from a research cruise conducted in 2017 (Table 1) and included. Dense sampling in mid‐Norway was complemented with 83 fish collected in Sula in 2013 (SUL13 in Figure 1). A total of 974 fish from south‐western and south‐eastern parts of the study region were collected during summer months (June–September) in 2013–2018 (Figure 1; Table 1). All fish were caught by trained research personnel or professional fishermen using fyke nets and pots, killed upon catch, and samples were taken immediately. Alternatively, killed whole fish were stored frozen until sampling in laboratory facilities. From each fish, a fin clip sample was taken for genetic analysis. When possible, biological data (length, weight and sex) were collected. The fish caught in Flatanger 2017 were also aged by counting annual growth increments in otoliths, following the procedure described in detail in Halvorsen et al. (2016). Age data from Flatanger 2016 were available from Faust et al. (2018).

**TABLE 1:** Corkwing wrasse sample information. Samples are arranged from north to south following the Scandinavian coastline

Sample location	Abbreviation^b^	County	Geographic group	Collection year	Geographic location^a^		Sample size
					Lat (N)	Lon (E)	
Flatanger	FLA16	Trøndelag	Mid‐Western	2016	64.53	10.75	105 (95)^c^
Flatanger	FLA17	Trøndelag	Mid‐Western	2017	64.53	10.75	365 (307)
Flatanger	FLA18	Trøndelag	Mid‐Western	2018	64.53	10.75	30 (30)
Stoksund	STO17#	Trøndelag	Mid‐Western	2017	64.04	10.07	1 (1)
Hitra	HIT17#	Trøndelag	Mid‐Western	2017	63.46	8.67	10 (10)
Edøya/Smøla	SMO17#	Møre og Romsdal	Mid‐Western	2017	63.32	8.22	13 (13)
Smøla	SMO18	Møre og Romsdal	Mid‐Western	2018	63.47	7.87	258 (245)
Tustna	TUS17#	Møre og Romsdal	Mid‐Western	2017	63.22	8.02	3 (3)
Kristiansund	KRI17#	Møre og Romsdal	Mid‐Western	2017	63.11	7.85	44 (43)
Averøy	AVE17#	Møre og Romsdal	Mid‐Western	2017	63.07	7.56	3 (3)
Sandøy	SAN17#	Møre og Romsdal	Mid‐Western	2017	62.82	6.60	3 (3)
Midsund	MID17#	Møre og Romsdal	Mid‐Western	2017	62.68	6.65	22 (21)
Ålesund	ALE17#	Møre og Romsdal	Mid‐Western	2017	62.45	6.33	40 (38)
Sula	SUL13	Møre og Romsdal	Mid‐Western	2013–2014	62.40	6.24	83 (77)
Måløy	MAL13	Vestland	South‐Western	2013–2014	61.94	5.12	5 (5)
Flora	FLO18	Vestland	South‐Western	2018	61.58	4.86	9 (9)
Os	OS14	Vestland	South‐Western	2013–2014	60.17	5.49	156 (134)
Austevoll	AUS14	Vestland	South‐Western	2013–2014	60.10	5.27	108 (91)
Austevoll	AUS17	Vestland	South‐Western	2016–2017	60.10	5.27	249 (233)
Sveio	SVE14	Vestland	South‐Western	2013–2014	59.52	5.51	182 (148)
Årdalsfjorden	ARD18	Rogaland	South‐Western	2018	61.20	7.57	14 (10)
Flødevigen	FLOD17	Arendal	South‐Eastern	2016–2017	58.42	8.76	110 (106)
Risør	RIS16	Agder	South‐Eastern	2016	58.72	9.20	41 (41)
Hvaler	HVA14	Østfold	South‐Eastern	2014	59.06	10.90	60 (60)
Marstrand	MAR16	Västra Götaland (Sweden)	South‐Eastern	2016	57.89	11.60	40 (40)

^a^ Given geographic location is an approximate midpoint for several sampling locations.

^b^ Samples marked with "#" were received as bycatch during research cruise in South Trøndelag and Møre og Romsdal counties.

^c^ Number in parenthesis is the number of samples genotyped successfully and used in analyses.

#### 2.2.2 Genotyping

Genomic DNA was extracted from fin clips using the Qiagen DNeasy Blood & Tissue Kit in 96‐well plates following the manufacturer's instructions. A total of 1954 unique individuals and 105 technical replicates were genotyped in four multiplexes for 106 SNPs. Loci that did not produce reliable clustering patterns were removed (*N* = 17). Loci and individuals with more than 20% missing data were removed, leaving 1766 individuals and 85 SNPs. Genotyping robustness was evaluated by calculating concordance between 79 successfully genotyped technical replicates, removing any locus with more than 2 discordant genotypes. One locus showed several discrepancies between genotypes (Figure S2) and was removed. The final data set consisted of 1766 unique individuals genotyped for 84 loci with a total of 2.9% missing data.

### 2.3 Statistical analysis

To ease analysing and discussion phases, samples were ordered from north to south along the coastline and grouped into larger geographic units defined as: “western” (Norwegian west coast), “southern” (Norwegian south coast and Swedish west coast) or as “mid‐western” (>62°N), “south‐western” (<62°N, <8°E) and “south‐eastern” (<60°N, >8°E) (Table 1). Unless otherwise stated, data manipulation and visualization of results was done using R v3.6.1 (R Core Team, 2019) and Rstudio v1.2.5019 (RStudio Team, 2019), mainly with Tidyverse packages (Wickham et al., 2019).

#### 2.3.1 Genetic diversity and divergence

Observed and expected heterozygosity for each locus across samples and over the three geographic regions was calculated using the R package diveRsity (Keenan et al., 2013). Deviations from expected heterozygosity (*H*e) were assessed by calculating *F*_IS_ according to Weir and Cockerham (1984). Deviations from expected Hardy–Weinberg proportions (HWE) were estimated with Exact test, and *p*‐values calculated according to the complete enumeration method and adjusted for multiple testing using Bonferroni correction (Louis & Dempster, 1987). Loci that deviated from HW proportions in more than half of the samples were subsequently removed. Weir & Cockerham's pairwise *F*_ST_ was estimated for each population pair as well as global *F*_ST_ across all samples. Statistical significance of *F*_ST_ values was assessed using Fisher's exact probability test with 5000 Monte Carlo replicates, followed by Bonferroni correction. The sample from Stoksund (STO17; see Table 1) only consisted of one fish and was thus excluded from all genetic diversity and divergence analyses. Distribution of variation between geographic regions, between samples within regions and within samples was investigated with AMOVA in the R package poppr (v. 2.8.6; Kamvar et al., 2014). Statistical significance for the variance components was obtained with 999 permutations using the ade4 package (v. 1.7‐16; Dray & Dufour, 2007).

#### 2.3.2 Individual‐based clustering and cline models

To estimate and visualize genetic differentiation among individuals, we applied two individual‐based clustering methods, STRUCTURE v.2.3.4 (Pritchard et al., 2000) and principal component analysis (PCA) in the R package ade4 (Chessel et al., 2004; Dray & Dufour, 2007; Dray et al., 2007). STRUCTURE is a model‐based Bayesian clustering method that uses a predefined number of K clusters to estimate the posterior probability of each individual's genotype to originate from each cluster. STRUCTURE analyses were performed for the data set including all samples using the default admixture model with correlated allele frequencies. To test the performance of different clustering algorithms, simulations were run with and without a priori location information (Hubisz et al., 2009). As it has been shown that uneven sampling can lead to erroneous clustering and that this problem can be alleviated by lowering the alpha parameter (Wang, 2017), values of 0.1, 0.33 and 0.5 were also tested besides the default value of 1. A total of 70,000 MCMC (Markov Chain Monte Carlo) repetitions were run and the first 20,000 were discarded as burn‐in. *K* was set from 1 to 6, and the number of iterations was set to 5. To determine the optimal solution for *K*, the StructureSelector software (Li & Liu, 2018) was utilized. The software summarizes results as the optimal Ln Pr(*X*|*K*) given by the STRUCTURE software and the ad hoc summary statistic Δ*K* by Evanno et al. (2005), which identifies the uppermost level of population hierarchy. Moreover, StructureSelector software produces and visualizes four alternative statistics (MedMed, MedMean, MaxMed and MaxMean) described by Puechmaille (2016). Results from the runs for the different values of K were averaged with CLUMPAK (Kopelman et al., 2015) using the LargeKGreedy algorithm and 2000 repeats. The second individual‐based clustering method (PCA) uses a multivariate exploratory approach that makes no prior assumptions about how many populations exist or boundaries between them. Allele frequencies were centred but not scaled and missing data were replaced by mean allele frequencies with the function scaleGen in ADEGENET (Jombart, 2008; Jombart & Ahmed, 2011).

Population divergence was also assessed with pairwise Pearson's correlation coefficient and scatter and density plots of allele frequency, estimated for each sample pair. Recent versus historical gene flow between south‐eastern and western populations was assessed using TreeMix (Pickrell & Pritchard, 2012), which infers the patterns of population splits and mixtures in the history of a set of populations. This method first builds a maximum likelihood (ML) phylogeny and subsequently models migration between branches to determine whether migration/admixture events improve the likelihood fit. Phylogenetic trees were calculated both with the default correction for sample size effects and without, as it in some cases can lead to overcorrection. First, a ML tree without migration events was constructed. After this, five more trees were built that included one to five migration events. Finally, variance explained by the different models, with different numbers of migration events, was calculated.

Cline analysis is used to estimate the shape, centre and width of the sigmoid curves generated by molecular, phenotypic or environmental markers, and to test for concordance and coincidence in these parameters between markers (Gay et al., 2008). Geographic cline analyses over a 1200 km transect between Flatanger (Norway) and Marstrand (Sweden) were conducted with the R package HZAR (Derryberry et al., 2014). The fifteen models implemented in HZAR were fitted to the allele frequency of every individual locus to determine the position, width and shape of clines over the geographic distance. A reference cline was built using STRUCTURE Q‐score for the total data set, and the best cline model was decided upon AIC scores. Clines were considered significantly displaced if the two log‐likelihood unit support limits of the cline centre did not overlap with the STRUCTURE Q‐score (Qb = 1– Qs). Temporal replicates were pooled, and sampled populations with small sample size (<10) were removed.

#### 2.3.3 Hybridization

In order to ensure high efficiency, accuracy and consequently power to detect true escapees and hybrids with the filtered data set of 84 markers, a second round of simulations was performed. The same procedure was used for both simulation and analysis as described above for the full panel of 106 SNPs. After simulations, the occurrence of escapees and hybridization along the Norwegian coast were investigated with the software NEWHYBRIDS. Analyses were done using the uniform prior option, default genotype proportions, and the burn‐in period was set to 50,000 and the number of MCMC sweeps after burn‐in to 300,000. Map visualization was done using the R packages shapefiles (Stabler, 2013) and mapplots (Gerritsen, 2018). The Wilcoxon rank‐sum test was used to compare mean age between hybrids and pure western genotypes, and between the years 2016 and 2017 (hybrids and pure western pooled in each year).

## 3 RESULTS

### 3.1 Genotype validation and power estimation

Individual genotyping was evaluated by comparing concordance between technical replicates. A total of 79 individuals were successfully genotyped twice with <20% missing data. Discordant genotypes were few and only present in two markers, one with 2 discordant genotypes and one with 15. Locus SYMME_00004618_13817, with 15 discordant genotypes was removed from further analysis, which resulted in a final data set of 84 SNPs (Figure S2).

Simulated hybrid data showed that the final panel of 84 SNPs maintained a high accuracy (>92%), efficiency (>83%) and power (>81) to assign all six hybrid classes (pure western, pure southern, F1, F2, western and southern backcross) at probability thresholds between 0.5 and 0.9. Furthermore, when pooling the F1, F2, western and southern backcrosses as a single hybrid class, these numbers increased to >97% accuracy, >95% efficiency and >95 power (Figure S3).

### 3.2 Genetic diversity

The overall diversity showed a similar pattern to what has been observed in previous studies, with much lower diversity south of the genetic break (Table S2). The mean observed heterozygosity ranged between 0.184–0.187 in south‐eastern samples and 0.315–0.413 in all western samples (*p* = 0.002). Similarly, allelic richness was significantly lower (*p* = 0.002) in south‐eastern samples (1.42–1.43) compared to the western samples (1.78–1.90). Differences in these diversity indices were statistically significant also when both western samples were compared separately with south‐eastern samples (*p* < 0.05 in all cases). Moreover, mean allelic richness was higher (*p* = 0.015) in south‐western samples (*A*_R_ = 1.83) than in mid‐western samples (*A*_R_ = 1.80), but no difference was observed for mean observed heterozygosity (*p* = 0.284). The majority of markers showed no deviation from HWE in any of the sampled locations and only two markers deviated significantly from HWE in more than six locations. Initial comparisons showed little to no difference in results when removing these two markers, and consequently, all markers were kept for further analysis. Overall, nine out of all sample populations deviated significantly from HWE. Eight of which showed heterozygosity deficiency (*F*_IS_ 0.017–0.06) and one heterozygosity excess (*F*_IS_ −0.012); all were observed in western Norway. Correlation statistics calculated by pairwise comparison of allele frequencies showed high positive correlation within each of the three geographic groups (Figure S4). South‐eastern samples had a negative correlation against all western samples with the exception of Årdalsfjorden. Density plots showed that Årdalsfjorden had intermediate allele frequencies in contrast to western and south‐eastern samples which were heavily skewed in opposite directions.

### 3.3 Population structure and individual assignment

~34% of the total genetic variation was distributed between the three geographic groups, and less than one percent between samples within these groups (Table 2). Consistently, pairwise *F*_ST_ estimates between sampled populations revealed an overall lower genetic differentiation within each of the three geographic groups than between them (Table S3). Within‐group differentiation was lowest in south‐eastern samples (mean *F*_ST_ of 0.0005 ± 0.0011), followed by the mid‐western samples (*F*_ST_ = 0.0054 ± 0.0052) and highest in south‐western samples (*F*_ST_ = 0.0120 ± 0.0146). When comparing divergence within and between the three geographic areas, the genetic differentiation within the western samples were order of magnitude lower (mean *F*_ST_ = 0.0216 ± 0.0119) than between the western and the south‐eastern samples (*F*_ST (mid‐west_vs_south‐east)_ = 0.5155 ± 0.0699 and *F*_ST (south‐west_vs_south‐east)_ = 0.4757 ± 0.0549). Of the western samples, Flatanger17 showed clearly lower differentiation towards the south‐eastern samples (mean *F*_ST_ = 0.3704 ± 0.0089), with all other pairwise comparisons ranging between 0.4106 and 0.6070.

**TABLE 2:** Analysis of molecular variance (AMOVA). Geographic groups and samples are shown in Table 1

Variance source	*df*	Sum of squares	Variance components	Percentage of variation	*p* Value
Between geographic groups	2	9770	9.1	33.9	0.002
Between samples within groups	22	623	0.2	0.7	0.001
Within samples	1741	30,758	17.7	65.5	0.001

**FIGURE 2:**
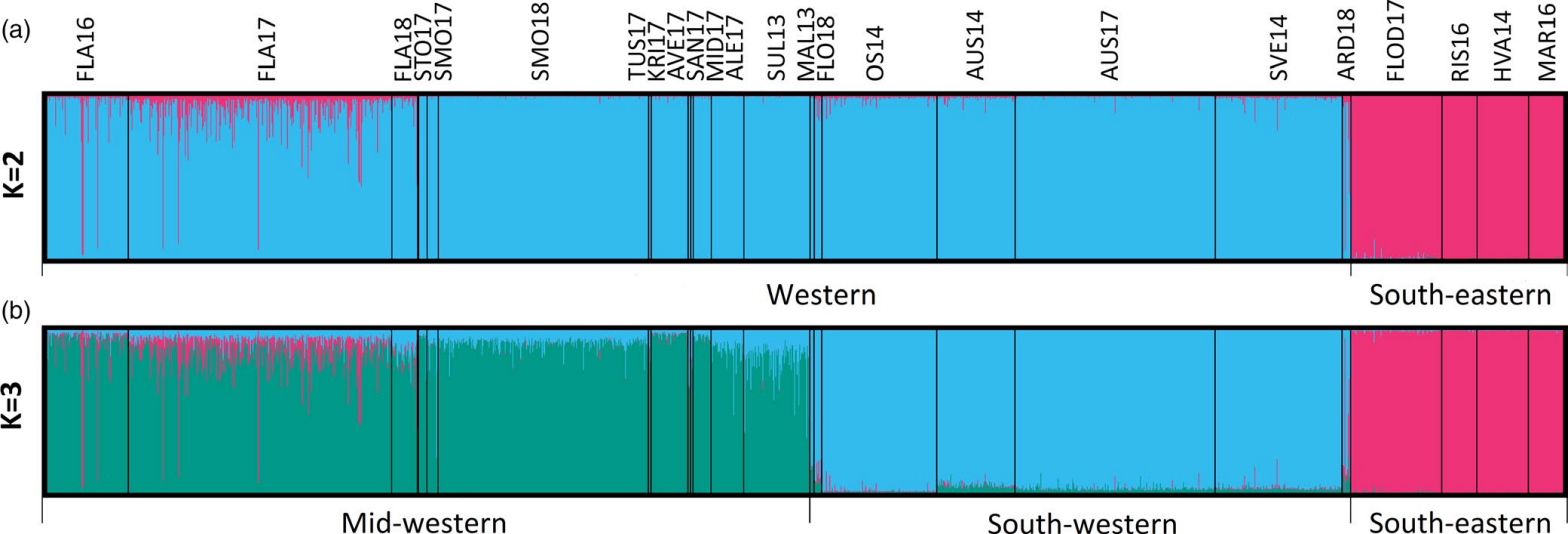
STRUCTURE cluster assignment of 1766 corkwing wrasse individuals based on 84 SNPs for *K* = 2 (a) and 3 (b) with sampling location given as a priori. Each vertical bar represents one individual and the colour the proportion of that individual assigned to the different genetic clusters. Individuals are sorted from North (left) to South (right)

In concordance with pairwise *F*_ST_ measurements, individual‐based clustering using STRUCTURE differentiated the south‐eastern cluster (pink) from the western samples (blue) (*K* = 2 in Figure 2). *K* = 2 was clearly supported as the highest level of population hierarchy by the Evanno method (Figure S5). Support for additional substructure was also evident: Adding one additional cluster (i.e. *K* = 3) splits western samples into two distinct clusters between Sula and Måløy implying an additional genetic break (green and blue in Figure 2; note that these clusters correspond to our Mid‐Western and South‐Western geographic groups, Table 1). Sampling location given as a priori clearly increased resolution power between the two western groups on an individual level for *K* = 3 (Figure 2; Figure S6a), but had little to no effect on the estimated admixture proportions with *K* = 2. Despite STRUCTURE gave clear clustering solutions with these two levels (*K* = 2 and 3) of population division, additional methods that were utilized favoured solutions for even higher levels of *K*s (4–5; Figure S5a,b). However, visual inspection of the corresponding bar plots (Figure S6a,b) shows that instead of creating new (vertical) separations between those well‐supported groupings of two or three, these clusters would merely build up additional layer(s) of difference and are thus likely technical artefacts depending on the model assumptions. Reducing the alpha parameter from its default value of 1 down to 0.5, 0.33 or 0.1 did not change the obtained results in any way (data not shown), suggesting that uneven sampling had no effect in the clustering analysis.

Assignment of individuals into genetic clusters with *K* = 2 was clear. When investigating individual membership coefficients (*q*), the vast majority of all fish (94.6%) had a *q* of ≥0.95 corresponding to their “own” geographic group (western or south‐eastern). However, six individuals from Flatanger and one from Årdalsfjorden in western Norway were assigned with a high proportion (*q* > 0.98) to the south‐eastern cluster. Moreover, nine individuals had roughly equally admixed genotypes (*q* = 0.4–0.6 to both clusters), 38 had moderate representation (*q* = 0.2–0.4) of the south‐eastern cluster in their genomes, and 40 rather low but still notable portions (*q* = 0.1–0.2), all suggesting varying degree of admixture between the clusters. When fish were assigned into three clusters (*K* = 3) instead of two, they were still highly concordant with their geographic origin (Figure 2; Figure S6c): Mid‐western individuals had a mean assignment of 0.899 (±0.058), south‐western 0.857 (±0.105) and south‐eastern 0.997 (±0.004).

**FIGURE 3:**
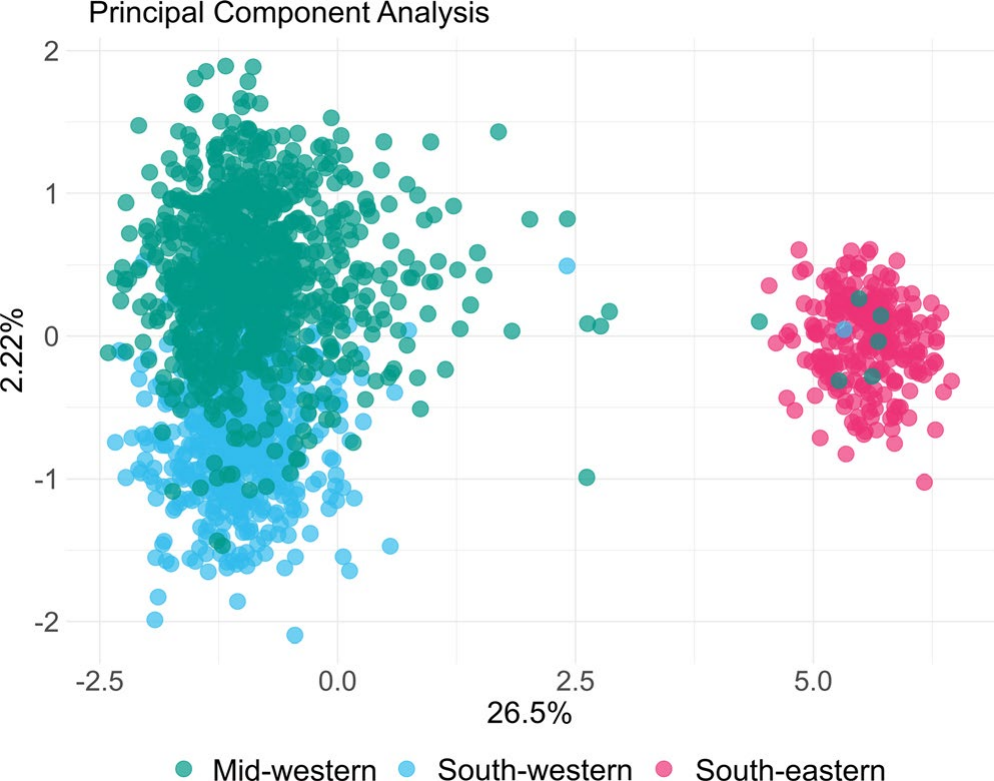
First (*x*‐axis) and second (*y*‐axis) component of a principal component analysis (PCA) on 1766 corkwing wrasse individuals based on 84 SNPs. The first component explains 26.5% of the total variation and the second 2.2%. Each point represents one individual, and colours represent the three geographic regions

The principal coordinate analysis (PCA) demonstrated a similar pattern as seen in the STRUCTURE cluster analysis (Figure 3, Figure S7). The first axis (*x*‐axis, accounting for 26.5% of the total variation) clearly separates south‐eastern samples (pink) from western samples (blue and green). The second axis (*y*‐axis, explaining 2.2% of the variation) separates the mid‐western samples (green) from south‐western samples (blue), but with a degree of overlap between the clusters. The seven individuals previously identified in the STRUCTURE analysis clearly cluster together with individuals from the south‐eastern cluster also in the PCA. Individuals previously identified as possible admixed in STRUCTURE analysis are also in the PCA located between the western and south‐eastern clusters.

The maximum likelihood (ML) tree calculated from TreeMix, which infers the patterns of population splits and mixtures in the history of a set of populations, clearly displayed the known genetic break and captured 98.3% of the variance (Figure S8a). South‐eastern samples clearly separate from western samples, both with and without migration. Mid‐western and south‐western samples grouped separately from each other for typologies with one or more migration events. The first and second migration events took place from south‐eastern samples to Flatanger and captured 99.2% and 99.6% of the variance, respectively. When zero to two migration events were modelled, Årdalsfjorden was inferred as an intermediate between western and south‐eastern samples, although closer to the western group. In the case of three migration events, Årdalsfjorden was placed among the western samples with migration from south‐western samples and the variance explained increased to 99.67%. The fourth and fifth migration events added migration among western samples, but did not increase the explained variance and were the only migration events not significant (*p* > 0.05) based on jack‐knife resampling. There were only minor differences between trees when not correcting for sample size, and thus, only the corrected models are shown. Inspection of the matrix of residuals (Figure S8b) indicated how well the tree model fits the data, in which residuals above zero represent populations that are more closely related to each other than in the best‐fit tree and are candidates for admixture, and negative residuals indicate that a pair of populations is less related than represented in the best‐fit tree. Positive residuals indicated that Flatanger and Årdalsfjorden might be admixed with south‐eastern samples and could benefit from additional edges. Flatanger residuals become closer to zero with both one and two migration events added and Årdalsfjorden with four migration events.

The reference cline based on the STRUCTURE Q‐scores fitted an optN model, with the centre situated at 799 km (787–1087) (Figure S9a). All the 84 loci fitted cline models with centres ranging between 706 and 1062 km (Table S4), and none of them was significantly displaced from the STRUCTURE reference cline (Figure S9b). This means that all loci showed a similar pattern of divergence. The cline centre is located close to the habitat break on the southwest tip of Norway.

### 3.4 Hybridization

**FIGURE 4:**
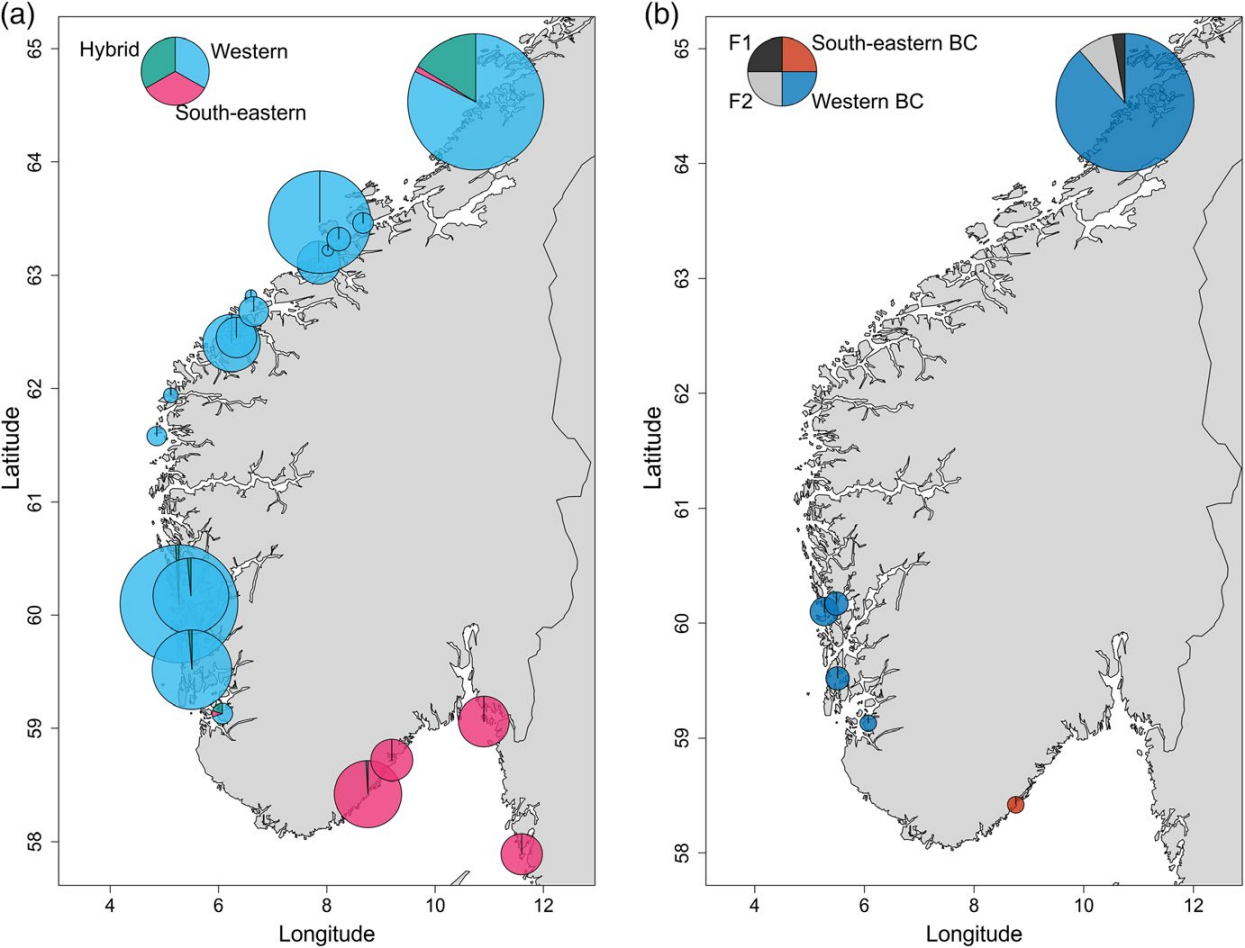
Proportion of individuals at each sampling site classified by Newhybrid analysis. (a) Left map displays individuals classified as western genotype, south‐eastern genotype or hybrid with >50% probability. (b) Right map displays the proportion of hybrids assigned to the different hybrid classes: F1, F2, backcross with western and backcross with south‐eastern. Pie sizes reflect the relative number of individuals

Samples were screened for potential hybrids using the software NEWHYBRIDS, which estimates each individual's probability of belonging to predefined classes (pure western, pure south‐eastern, F1, F2, western backcross and south‐eastern backcross). Of the 1766 individuals analysed, all of them could be assigned with a probability >50% to be either pure western (blue) or pure southern (pink) or hybrid (green) (Figure 4a). When distinguishing between the different hybrid classes (F1, F2, backcross 1 and backcross 2), all but one individual could be assigned with a probability >50% (Figure 4b and Figure S10a). When increasing the probability threshold to >80%, 1715 individuals could still be assigned to the different hybrid classes. Among the western samples, seven individuals had a very high probability (>90%) to be of pure‐eastern origin, six in Flatanger and one in Årdalsfjorden. The majority of all potential hybrids could also be found in Flatanger where 70 individuals had more than a 50% probability to be F1, F2, western backcross or south‐eastern backcrosses (Figure S10b). In all other western samples, only nine individuals could be identified as potential hybrids, all of them as western backcrosses. The age distribution did not differ between hybrids and pure western genotypes in the Flatanger in either 2016 or 2017 (Figure S11; Table 3; *W*_2016_ = 314, *p* = 0.7; *W*_2017_ = 5642, *p* = 1). The two oldest individuals were classified as hybrids (aged 8 and 9 years), while the oldest individual with pure western genotype was 7 years old. Of the six individuals classified as of pure southern origin, all were larger than 175 mm (minimum size limit in the fishery for corkwing is 120 mm) and between 2 and 4 years old (Table 3).

**TABLE 3:** Summary of sex ratio, age and length of corkwing wrasse sampled in Flatanger 2016 and 2017 for each hybrid class

Class	Year	*N*	% females	Mean age (range)	Mean length, mm (range)
Hybrid	2016	7	57	2 (2–3)	150 (107–178)
Pure Western	2016	84	41	2 (2–5)	153 (112–208)
Pure South‐eastern	2016	3	33	2 (2–3)	176 (172–185)
Hybrid	2017	55	60	3 (1–9)	161 (95–215)
Pure Western	2017	205	57	3 (1–7)	162 (100–200)
Pure South‐eastern	2017	3	100	4 (3–4)	186 (177–195)

## 4 DISCUSSION

We developed and implemented a panel of informative SNPs to quantify the proportion of escaped and hybridized corkwing wrasse with a southern origin in middle and western Norway, where translocated cleaner fish are extensively used for parasite control. The panel of 84 SNPs, which can detect escapees and hybrids with a power >0.95, identified a total of 7 escapees and 79 potential first‐ and second‐generation hybrids among 1519 analysed fish on the Norwegian west coast. Most of these were identified in samples from the northernmost part of the species distribution in mid‐west Norway, which also represents the main area of import from southern latitudes. On the basis of these results, and, given the large documented genomic differences between corkwing wrasse between the export and import regions, current practices should be revised to take the natural population structure into account and avoid moving individuals between genetically differentiated populations.

### 4.1 Genetic differentiation among wrasse populations

The panel of informative SNPs was developed to identify genetic differences between source populations in southern Norway and Sweden versus local wild wrasse populations in receiving areas in western Norway. In concordance with previous studies (Blanco González et al., 2016; Faust et al., 2018; Mattingsdal et al., 2020), we found strong genetic divergence between corkwing wrasse on the west and south coast of Norway. All SNPs showed a similar pattern of divergence with a cline centre located close to the habitat break, on the south‐western tip of Norway (Figure 2, Figure S9).

In addition to the previously documented main genetic break located at south‐western Norway, a second weaker break in genetic structure was detected at ~62° N on the Norwegian west coast. Blanco González et al. (2016) described a pattern of moderate isolation by distance among corkwing samples collected along the west coast of Norway and found their northernmost samples (Vestnes 62.65°N and Smøla 63.32°N) to be distinct from south‐western samples. However, few studies compare samples north of 60.2°N and none have included samples from the areas between 60°N and 62.4°N. Despite the relatively few individuals (*N* = 14) available from this region in the present study, all of them were clearly clustered within the south‐western group, indicating that there could be a stronger genetic discontinuity in this region than previously indicated. However, the markers used here are not ideal to resolve genetic population structure in this region as they were selected to distinguish south‐eastern from western samples. It is therefore not possible to disentangle the nature of this second break, that is whether selection or neutral processes are at play.

### 4.2 Extent of escapees and hybridization

Hybrid analysis identified a total of 7 individuals as potential escapees and 79 as potential hybrids on the Norwegian west coast. The majority of these individuals were caught in Flatanger, Trøndelag (6 potential escapees and 70 hybrids; in total 17.6% of samples from Flatanger), which is also the northern range of the species current natural distribution. It is noteworthy that besides fish identified as escapees or hybrids, majority of samples from Flatanger had some genetic resemblance to south‐eastern population (mean *q* score of 6.5%; Figure 2), while no such pattern was observed for fish sampled elsewhere in mid‐western Norway (*q*‐score_mean_ <0.1%). The only other pure south‐eastern individual was found in Årdalsfjorden, <60 km from the sandy beaches in Jæren, where the main genetic break has been previously identified (Blanco González et al., 2016). Out of the 10 individuals successfully genotyped in Årdalsfjorden, one was identified as of south‐eastern origin and two as hybrids. In all other south‐western samples, we found no more than one or two potential hybrids. Given the proximity to the genetic break (Mattingsdal et al., 2020), this relatively large fraction of individuals of south‐eastern descent in Årdalsfjorden could potentially be explained by natural migration and gene flow across the break. Cline analysis put the cline break very close to Årdalsfjorden which could suggest that there is a possibility for natural gene flow from south‐eastern populations. TreeMix analysis also showed that Årdalsfjorden could be a stepping stone between south‐western and south‐eastern populations. We found Årdalsfjorden to have intermediate allele frequencies, in contrast to western and south‐eastern samples which were heavily skewed in opposite directions (Figure S4), which could be an indication of an admixed population. Finally, given the high population densities in the region, it is likely that most cleaner fish used is sourced locally which would suggest that admixture in Årdalsfjorden could be a result of natural migration. Besides Flatanger, we did not detect any potential escapees or hybrids in any other parts of the Trøndelag county or its neighbour Møre og Romsdal county, despite the relatively large number of fish sampled and the fact that these are also high‐density salmon farming areas. The potential explanations for this striking result are discussed below.

**FIGURE 5:**
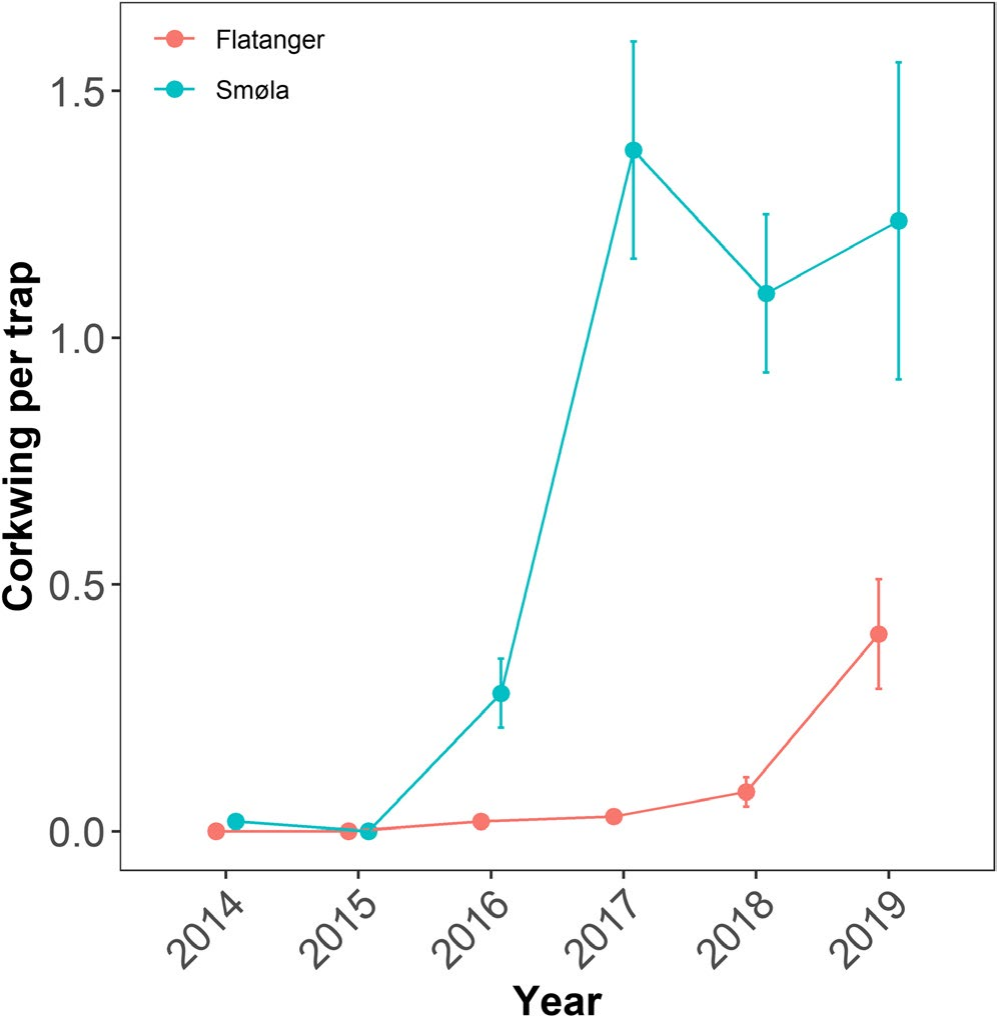
Development in raw catch‐per‐unit effort (CPUE) for corkwing caught in commercial trap fishery (one fisher per location). CPUE is calculated as the total N corkwing caught, divided by the total number of traps sampled in each year. Error bars show ±*SE* of the mean

The lack of escapees or hybrids detected in Møre og Romsdal compared to Trøndelag could be explained by a combination of different factors, for example: (1) corkwing wrasse only expanded into Trøndelag recently and the population size is therefore small (Maroni & Andersen, 1996), and thus, escapees and hybrids are easier to detect, (2) smaller native populations make it easier for escapees to establish due to less competition (Glover et al., 2012; Heino et al., 2015; Rhymer & Simberloff, 1996) and (3) there is less import from the south‐eastern population to Møre og Romsdal and/or less individuals are escaping. The abundance of corkwing in mid‐Norway (i.e. Trøndelag and Møre og Romsdal counties) has only recently increased, suggestively indicated by the catch‐per‐unit effort (CPUE) data from fishermen in this region (Figure 5). In Smøla, the catch rates increased in 2015 and have levelled out after 2017. The population in Flatanger appears to still be in an early phase of establishment and was virtually absent from catches until 2018 (Figure 5). We found hybrid individuals as old as nine years in Flatanger, which implies that escape and hybridization have been ongoing for at least a decade in this area, coinciding with the intensification of the national wrasse fishery in 2009–2010 (Figure S12). Earlier work has shown that corkwing in western Norway can reach twice the age (8 years) of corkwing wrasse from Skagerrak (4 years) (Halvorsen et al., 2016; Uglem et al., 2000). The older hybrid found in this study suggests that the introduction of southern material may not impact the longevity of local western populations. However, this and other aspects of hybrid fitness still need to be investigated.

A recent study found potential fitness differences between corkwing wrasse in southern and western Norway (Blanco Gonzalez et al., 2019), suggesting the possibility for local adaptation. Even weak to modest negative selection against translocated genotypes would reduce the frequency of hybrids in a large population (Castellani et al., 2018), such as in Smøla. However, in a smaller population, such as in Flatanger, selection would be less effective (Allendorf et al., 2013; Bridle & Vines, 2007).

Overall, more cleaner fish are used in aquaculture in Trøndelag than in the county below, Møre og Romsdal. However, during two of the four years for which data are available (2016 and 2017), almost twice the amount of corkwing wrasse were used in Møre og Romsdal compared to Trøndelag (Norwegian Directorate of Fisheries, 2019; Figure S12). Given the higher population densities in Møre og Romsdal (Figure 5), it is likely that more fish is sourced locally than in Trøndelag. This is also corroborated by import from Sweden. Since reporting started in 2017, more than three times as many corkwing wrasse have been transported to Trøndelag compared to Møre og Romsdal (Figure S13).

During 2017 and 2018, an average of less than 0.4 million corkwing wrasse were imported from Sweden per year. During the same years, an average of 7 million wild corkwing wrasse were used in Norwegian aquaculture. Thus, Swedish imports constitute less than 6% of corkwing wrasse used as cleaner fish in Norwegian aquaculture. However, source and destination of corkwing wrasse caught in Norway are not reported. This makes it difficult to estimate how many of the corkwing wrasse used in commercial salmon farming originates from the southern coast of Norway, as opposed to local sources. Catch numbers suggest that on average 20% of wild‐caught cleaner fish are caught off the southern coast of Norway annually, but most years less than 1% of all cleaner fish is being deployed in the southern region (Norwegian Directorate of Fisheries, 2019). Given the current lack of reporting, it is not possible to estimate where southern corkwing are transported to. The lack of reporting also complicates potential estimation of the number of escapees. Although all Norwegian fish farms are obligated to report escaping fish, currently this is only applied to the target species being farmed (i.e. salmon).

### 4.3 Implications

The effects of translocation between genetically distinct populations are difficult to predict and depend on many factors. Direct escapees can cause ecological effects and transmit novel diseases and pathogens. If hybridization occurs, genetic effects can also be anticipated as has been observed for a wide variety of traits in the case of domesticated salmon escapees in wild populations (Glover et al., 2017). Several escapees and backcrossed individuals were identified in the northernmost locations sampled. In addition, the Structure analysis indicates that in Flatanger, the majority of the investigated individuals show admixture (Figure 2; Figure S6c). This means that a notable fraction of the population gene pool has a southern origin and that more permanent genetic changes (i.e. introgression) could also have taken place. In contrast, we did not detect such hybridization in for example Smøla despite frequent and abundant translocation of fish from south to this region. Studies of genetic effects of stocking have shown that large and/or well‐connected populations seem to be relatively resistant to introgression (Bruce et al., 2020; Rougemont et al., 2019), likely because of simple dilution effect and selection being more efficient in large populations. While environment (Bruce et al., 2020) and population dynamics (Meirmans et al., 2009) also play some role, the level of admixture and hybridization success seem to be mostly dependent on stocking intensity and timing (Harbicht et al., 2014; Létourneau et al., 2018; Wringe et al., 2018). Thus, if the relative proportion of escapees is large and is still ongoing, the impact is likely to be greater and last longer (Castellani et al., 2018; Wringe et al., 2018).

The consequences of hybridization in the northern edge population are hard to predict but given the considerable difference in important abiotic factors between this region and southern Norway and Sweden, inadvertently translocated individuals are likely to be maladapted and have lower fitness in the recipient populations. For example, the onset of the reproduction is affected by photo‐period and temperature (Stone, 1996). If a genetic component is involved as well, it is possible that hybrids may initiate spawning at an unfavourable time‐of‐year (DeRito et al., 2011), resulting in reduced survival of offspring. Furthermore, genetic differences may include life history, physiological and morphological traits that negatively affect fitness, thus reducing the overall population viability, as well as the capacity to naturally expand further north as the environment changes. Future work in this direction should assess phenotypic differences between individuals with native and southern origin and ideally do field studies comparing fitness between these groups (e.g. tagging experiments, field observations of reproduction) and/or controlled common garden experiments to assess differences in phenotypic plasticity and physiology. Such studies have unequivocally demonstrated lower fitness of domesticated Atlantic salmon offspring in wild populations (Skaala et al., 2012, 2019).

The recently established Flatanger population is on the leading edge of the current species range and thus likely preselected to extremities of the species reaction norm, carrying favourable genetic material also for future range expansion northwards (Gibson et al., 2009; Moffett et al., 2018; Walters & Berger, 2019). However, the ongoing asymmetric gene flow from southern genotypes could influence further adaptation and range expansion (Kirkpatrick & Barton, 1997), and possibly even pose a risk for the whole population viability in future (Ghosh et al., 2012. Investigating if local adaptation of the admixed populations in the northern part of the species distribution is affected would require experimental studies. Given the predicted climate changes of warmer sea temperatures, populations at the northern edge of species distributions should be prioritized. We thus argue that any evaluation of the risk of translocation should not only include wrasse imported from Sweden but also the existing knowledge of genetically distinct populations within Norway. The lack of documentation regarding the source and destination of cleaner fish transported within Norway is a big obstacle to assess and address the challenge of escapees.

## 5 CONCLUSION

We developed a SNP panel with the ability to detect corkwing wrasse translocated from Skagerrak‐Kattegat to the Norwegian west coast as well as first‐ and second‐generation hybrids. Using this panel, we found that the geographical extent of escapees and potential hybrids is largely limited to areas at the northern edge of the species distribution where the number of escapees and potential hybrids may constitute up to 20% of the population. These results provide important knowledge, a baseline of the geographical extent and magnitude of hybridization, and a tool for management and monitoring of the future use of corkwing wrasse as a cleaner fish for parasite control. Moving genetic material between distant populations could alter the genetic composition, erode population structure and potentially result in loss of local adaptation, hampering the species expansion. The translocation and number of escaping cleaner fish are today poorly documented and regulated and the ecological consequences of escapees and hybrids remain unknown for this and other wrasse species. Recording the numbers, source and destination of wild‐caught cleaner fish on a per‐farm basis, as well as reporting escapees at the end of a season, would improve the ability to assess current and future risks associated with the use of cleaner fish for parasite control. Finally, based on the results of this and studies of other species of wrasse (Jansson et al., 2017; Seljestad et al., 2020) with similar challenges, we emphasize the need to reassess the current management practices involving massive translocation of nonlocal wild wrasse.

## CONFLICT OF INTEREST

None declared.

## Supporting information

Supplementary Material

## Data Availability

The data that support the findings of this study are openly available in the Institute of Marine Research electronic archive at: https://hdl.handle.net/11250/2731320. All raw sequences that were used to develop the SNP markers are available on NCBIs Sequence Read Archive (BioProject PRJNA702627; Faust et al., 2021).
