# Supplementary material for: Not that clean: Aquaculture‐mediated translocation of cleaner fish has led to hybridization on the northern edge of the species' range
Source: Evol Appl. 2021 Mar 29;14(6):1572–87. doi: 10.1111/eva.13220 (PMC8210792; doi:10.1111/eva.13220)

**Supplementary Data for the article:** “Not that clean: Aquaculture-mediated translocation of cleaner fish has led to hybridization on the northern edge of the species’ range”.

**Supplementary tables**

Table S1. Assay information for all 106 SNP markers. The 84 markers included in the final analysis after filtering are marked in bold. Fst is the estimated pairwise Fst between Austevoll and Risør based on 2b-RAD sequence data.

| **Assay** | **SNP_ID** | **2nd-PCRP** | **1st-PCRP** | **UEP_SEQ** | **Fst** |
| --- | --- | --- | --- | --- | --- |
| W1 | **SYMME_00001019_259056** | ACGTTGGATGTTGGGAGAACTACGACTCAC | ACGTTGGATGTACTGAGGGTTTGCCTCTTG | CTCACATGCCAACACAC | 0.4064 |
| W1 | **SYMME_00000351_687296** | ACGTTGGATGGGTAAAAGATTTAAAAGGCCC | ACGTTGGATGCCGGCTAAAAATAAAGTCTG | AAAAGGCCCTTGTATCA | 0.4223 |
| W1 | **SYMME_00023818_156483** | ACGTTGGATGGGCAAGTCCTGTCAGGAAAC | ACGTTGGATGAGAGAATGGATCAGTCGAGG | ATTGACAGGACGAGGAG | 0.4703 |
| W1 | **SYMME_00006679_62104** | ACGTTGGATGACACCAACGTGGGACTTATG | ACGTTGGATGTCACTTGGCTTGGCCAAAAC | GGGACTTATGTCACATGC | 0.5827 |
| W1 | **SYMME_00007129_47437** | ACGTTGGATGGAGATGAGCCCAGAGATTAC | ACGTTGGATGGAAAAAGAGGACGACCTTGC | CTTTAACCGACCTTCCAAA | 0.5127 |
| W1 | **SYMME_00023738_421399** | ACGTTGGATGACTGGGACGAGAAACCTTTG | ACGTTGGATGTCAACCGCTGATTGTGCAAC | CTGATCACGCTCGACTACT | 0.5271 |
| W1 | **SYMME_00004764_285875** | ACGTTGGATGTCAGCACTTTGAGCAGAGAC | ACGTTGGATGTCCGTCTGGATCACGATGAG | TGGTGAACGTTTTGTAGAC | 0.492 |
| W1 | **SYMME_00004729_285551** | ACGTTGGATGAGGTGTAGTTATGAGGGAGC | ACGTTGGATGATGACTGCCCCGTGTTCCTG | caAGACAAGCTGTCCTCGAT | 0.861 |
| W1 | **SYMME_00000109_1247032** | ACGTTGGATGATTCTCTCCACTGCAGACAC | ACGTTGGATGTTTTGTGCAGACAGAAGGTG | ggaAGACACGAAAATCATGC | 0.8103 |
| W1 | **SYMME_00023453_96630** | ACGTTGGATGTAAAACGCCTCTACATGCCC | ACGTTGGATGTTCGCCACGTTAATGTGAGG | tCCTCTACATGCCCCGCACGA | 0.7152 |
| W1 | **SYMME_00001278_977669** | ACGTTGGATGATTACAGATCAGTGGGTGCG | ACGTTGGATGGAGCCTGTTAGCTTAGCTTC | atATCAGTGGGTGCGACCTCA | 0.562 |
| W1 | **SYMME_00023225_405765** | ACGTTGGATGGCTTGAAGAGATTGTGACTG | ACGTTGGATGGCTACTCAAACAAAGTGACTC | ACTGAAGTATTGATTGGGAAA | 0.6879 |
| W1 | **SYMME_00006025_40517** | ACGTTGGATGAAGGAAGACAACTGCTCAGG | ACGTTGGATGAGACAGTGCTATCAGACAGG | cgctAAATTGCTGCCTACCTAA | 0.4694 |
| W1 | **SYMME_00023574_386442** | ACGTTGGATGTCTACCGTCATCGGTGAAAC | ACGTTGGATGGCACTTGGACATTAGCACTG | cTATCTGTTGGTTACAGCAGAC | 0.414 |
| W1 | **SYMME_00002115_94621** | ACGTTGGATGGCCCGTCAGCAAGTTTTTCG | ACGTTGGATGGCTGCGTCAGTCAATTTATC | ccCAGCAAGTTTTTCGAGGTAA | 0.4805 |
| W1 | SYMME_00000376_965994 | ACGTTGGATGCAGAGACCCCGAGTATTTAG | ACGTTGGATGGTTGGCTCGAGGTGAATTCT | gttgAAGTTAAGTCAGAGTGGG | 0.4241 |
| W1 | **SYMME_00023529_208893** | ACGTTGGATGTGAACTGCAAATGGCTTTGG | ACGTTGGATGTACCTTTCTACGGCAGCATC | GGAAATAAAGCAAAGACAAACAC | 0.4018 |
| W1 | **SYMME_00001181_204509** | ACGTTGGATGCAGAGTATTTACTTGATGGC | ACGTTGGATGGAACCCGAGAAGAAATGACC | tcTTTACTTGATGGCACCCGATTC | 0.4056 |
| W1 | **SYMME_00002662_67836** | ACGTTGGATGGGAAAGCAGTAGCTTCTTAC | ACGTTGGATGTACACAGAGATCACGAGATG | agtcCACTCACGTTGTTGATCATC | 0.6219 |
| W1 | **SYMME_00003318_375819** | ACGTTGGATGCGTCACTCGTGATTCTCTTC | ACGTTGGATGCTGGTTTCATGGCAAGCCTC | tttCTTCCCTTTTTTTTTTCTCGCT | 0.5313 |
| W1 | **SYMME_00023380_3836** | ACGTTGGATGAGGCGATGTCGAGAGAGAAG | ACGTTGGATGTGGCGCGGATGTAAATTCTC | gggaGACGACGAACGACGTGCAAA | 0.5307 |
| W1 | **SYMME_00002116_350960** | ACGTTGGATGTTTGTGTGCGATAGAGAGCC | ACGTTGGATGATGGATGCAGTGGATGTAAG | tcCTGGCAGATGTGTCGATCAACGC | 0.5366 |
| W1 | **SYMME_00023319_1170712** | ACGTTGGATGCAGTGCATTGATTCACGCAG | ACGTTGGATGATTGACAGCAAGGTGACTTC | tcagTGATTCACGCAGGAAATTCGC | 0.614 |
| W1 | **SYMME_00010158_75346** | ACGTTGGATGGGCCTCCCGTATAATAATGG | ACGTTGGATGAGTGAAGTTTGCGATCGAAG | taaCTTTTGTGTCTTTAGCCTCTCTC | 0.4214 |
| W1 | **SYMME_00023197_121823** | ACGTTGGATGCACTTCTTGTTGCAGAGTTC | ACGTTGGATGTTCGTTTGGAATCCCGATGC | gaacCAGTTGACGGCTTTAAGATAAA | 0.5189 |
| W1 | **SYMME_00023145_2392136** | ACGTTGGATGCTGGTCGATCATAAAACGTTG | ACGTTGGATGAGCCCAACCGATATGTTTGC | ggggTCAGGACACTACTGGTGTATCT | 0.5573 |
| W1 | **SYMME_00013619_22969** | ACGTTGGATGTAGAGTCGGTCTCCTCTAAC | ACGTTGGATGCACAGGAAGTGACATCACTC | gtcgCCTCTAACGAGGTCACTGCCCCC | 0.5047 |
| W1 | **SYMME_00012017_14751** | ACGTTGGATGGGTGGGATTTGAACCCGCAT | ACGTTGGATGCTGCACGAAAAATCAATCTG | ccttTACGCACAGACCTCGAGACTACT | 0.5697 |
| W2 | **SYMME_00001242_101541** | ACGTTGGATGTCCTCACCTATCCATACACC | ACGTTGGATGGCAGGTCACAACAACCAATG | TCCACACCTCTGCACAC | 0.4051 |
| W2 | **SYMME_00002850_26023** | ACGTTGGATGCTTTAGGTCGTCTGCAGAAC | ACGTTGGATGAACCTGGAATTTAGCGCCTG | TCTGCAGAACGCGCTAC | 0.704 |
| W2 | SYMME_00023206_68332 | ACGTTGGATGAAGAAGCAGGTGCAGCATTG | ACGTTGGATGGTTATTGTGGACATGTCCCG | GTGCAGCATTGCAGAAA | 0.6112 |
| W2 | **SYMME_00023953_207211** | ACGTTGGATGAATCGTGACAGCAGCTTCTC | ACGTTGGATGACTTTGCTGTGGAGACCTTG | CTGCAATCGCCATCCCTC | 0.4662 |
| W2 | **SYMME_00023262_816209** | ACGTTGGATGACCATCAGATGAGAAAGGCG | ACGTTGGATGACAAAGCTGTGCGTATAGCC | CTATATATGCGCAAGCTG | 0.5592 |
| W2 | **SYMME_00007078_108905** | ACGTTGGATGTTGCAGAAGCTCTCTCCATC | ACGTTGGATGTACTGTATTAACCCGGCCTC | GCTCTCTCCATCCTCCGCA | 0.5773 |
| W2 | **SYMME_00008787_21327** | ACGTTGGATGAGTGGTTGCGTGCACAGTTG | ACGTTGGATGGAGACGGATGAAAAGGTCAG | ccTGACCTTTCGATGACCT | 0.6584 |
| W2 | SYMME_00023260_787080 | ACGTTGGATGAAGAGAGTGATCCATGTGGC | ACGTTGGATGGAAAGTAAAGGATGTGACCC | CATAATGCTCGTGTCTGAT | 0.5477 |
| W2 | **SYMME_00000097_411069** | ACGTTGGATGGTTCATCTGTACTCTGTACC | ACGTTGGATGCCCAGAATCTGACATTTGTG | TGTACTCTGTACCTGCATCA | 0.4256 |
| W2 | **SYMME_00000375_42132** | ACGTTGGATGAAAATTCCTCAGGTGCAGGG | ACGTTGGATGTCACTTCAATGACCTTTAC | AAAGCAGAGCAAGCTAATCG | 0.5299 |
| W2 | SYMME_00003351_308412 | ACGTTGGATGGTGTGTGTGAAATGATGGG | ACGTTGGATGTTTATCGGCTGGCAGATTA | ccAAAATGACAACTAGACACC | 0.4106 |
| W2 | **SYMME_00001599_151719** | ACGTTGGATGGCCTAATCACATTGAGAAGC | ACGTTGGATGTGGATGTCGTCTTAGTGTCG | AGACAAAAATGCTCCAAGTGA | 0.4799 |
| W2 | **SYMME_00007721_148340** | ACGTTGGATGGTCAGCGAGGCGAGTAAGAT | ACGTTGGATGCCCACAGGGTAACAACAATT | CGAGGCGAGTAAGATGCAAAT | 0.4656 |
| W2 | **SYMME_00003452_329369** | ACGTTGGATGCACTATGATGCCTGCTCAAG | ACGTTGGATGCACAAATAACTGTCTTCGGC | ccCATCCATATGTCAACGGTAA | 0.4033 |
| W2 | **SYMME_00011099_16235** | ACGTTGGATGACTGAGGGCCAGTTGTAAAG | ACGTTGGATGAAACCCGGGAGACGAATGAG | aaaTCGATAAAACTGCCTCTCA | 0.4955 |
| W2 | **SYMME_00005187_201142** | ACGTTGGATGGATGGCAGGACTTTTTCAGA | ACGTTGGATGTGCATTCAGCGTTGGCATTT | ggGCTTAACTGTGACGAATATG | 0.4163 |
| W2 | **SYMME_00001871_333256** | ACGTTGGATGTTGTCCCCAGCATGTGATAC | ACGTTGGATGTGATTTCACATGTTGGGCAG | tcATCAAAACAGTTGTGCTAAAT | 0.4327 |
| W2 | **SYMME_00002972_463186** | ACGTTGGATGGCCGACGGCAAATCAAAAAC | ACGTTGGATGACCAGAATCAGCAGCGATAC | ttACGAAGCAGAGAAGTCGTCCG | 0.434 |
| W2 | **SYMME_00006969_69288** | ACGTTGGATGCAGGCAGATTCAATCTGACG | ACGTTGGATGCTGTCCACTTTTTGGTTTGC | gaaAGAACAGATCTGAGTGCTAC | 0.6054 |
| W2 | **SYMME_00003129_299972** | ACGTTGGATGGATCTGGGCTCAGTTCATTG | ACGTTGGATGTCCAAGTGCTGATCTAGGAG | ggGGTTACGGTAAAGATGCTGAA | 0.5943 |
| W2 | **SYMME_00023242_777958** | ACGTTGGATGTGTGAGAATTTACTACGTGC | ACGTTGGATGCTTTTGCTTCGAGGTCTGTG | ccTGCCAAATACTGCATTTTTTTT | 0.4155 |
| W2 | SYMME_00003544_13076 | ACGTTGGATGGAGAAGACAGTTTTCACCCG | ACGTTGGATGTTAGTGGCGACATTCTCCTC | cttCTCGTATTTTAGAGTCTTAACG | 0.5974 |
| W2 | **SYMME_00024144_124879** | ACGTTGGATGTCCGAGAGTGTTTGTGACAG | ACGTTGGATGTCAGTGCTGCTAAATAGACC | cGTTAGCAATGCAATCGAATTTTTT | 0.4797 |
| W2 | **SYMME_00024110_27315** | ACGTTGGATGTTCTCCTTTTGGCGTCACTG | ACGTTGGATGAGCAACGTTAAGACCAGCAC | gTAGTTGACGCGAGATTAGTGCAAA | 0.842 |
| W2 | SYMME_00002174_27173 | ACGTTGGATGAAACAAAGAGGAAAGCAGGG | ACGTTGGATGCAGACTCAGAAGAAGTCAGC | ggGAGGAAAGCAGGGGATTCGTCTT | 0.6256 |
| W2 | **SYMME_00024098_237080** | ACGTTGGATGATAAGGGCGAGCTATTCGTG | ACGTTGGATGGAGATTTCAGGCTTGGTCAG | gTAAGGGCGAGCTATTCGTGGGCGGC | 0.4465 |
| W2 | SYMME_00023208_352029 | ACGTTGGATGACGCAGCGTGCAAATCTCTC | ACGTTGGATGCCCTTTCCATTCTTCTCCTC | cCGCAGCGTGCAAATCTCTCGCCTGAA | 0.4942 |
| W3 | **SYMME_00023308_56447** | ACGTTGGATGGAGCAAGCCGACAGAATAAG | ACGTTGGATGCTCTACTGGTTGGCTGAAAG | CGACCCCCATGCACTCT | 0.5016 |
| W3 | **SYMME_00001073_633173** | ACGTTGGATGCCTTCGCATCACTCTTCGAC | ACGTTGGATGTGGCAGAAGCAGCATTTTCG | CCAGACAAGCTGCCCAG | 0.4467 |
| W3 | **SYMME_00002674_685013** | ACGTTGGATGCATTTCCATCTGCAGGCAGG | ACGTTGGATGGTCTTAGGTCTTAGCATGTG | CTGCAGGCAGGTCGACA | 0.5584 |
| W3 | **SYMME_00003351_50140** | ACGTTGGATGGAGGCAATAAACAAGCAGAC | ACGTTGGATGCTCAGTTTGATCACTCGTCC | ACAAGCAGACAAGTCGG | 0.7481 |
| W3 | **SYMME_00002315_632632** | ACGTTGGATGGCAGTCCACGTTCTTATGTC | ACGTTGGATGCGATTACACAAAGCACTGGG | TCAGGAGAATGGCGATGG | 0.4198 |
| W3 | **SYMME_00001760_736838** | ACGTTGGATGAGTCCACTGTGTTTGTGCTG | ACGTTGGATGAGGAAATGGAGGCATGGAAC | aTAATTGCACCATTCTCGG | 0.4366 |
| W3 | SYMME_00004618_13817 | ACGTTGGATGTACCCACTGCAGTTGTCTCG | ACGTTGGATGAATCCAGCCCTCATTTTGCC | ctAGTTGTCTCGTCTGATG | 0.5964 |
| W3 | SYMME_00000376_1475703 | ACGTTGGATGAGTCGAGTCTGGTTCATCAG | ACGTTGGATGGTTAGTCCTGATGCAGTGTG | CTTACCATTTGAGGTTGAG | 0.5081 |
| W3 | **SYMME_00010876_30588** | ACGTTGGATGGATTCCGTCGACTCGTCATC | ACGTTGGATGTAAGGAAACTACGGAAGCCC | GGGGAGAACGAAGGCGAGC | 0.4426 |
| W3 | SYMME_00000230_1023038 | ACGTTGGATGAGGAAGGTAGTCACAGGTTG | ACGTTGGATGAATAGCGGTCTCTTGTCAGC | AGTCACAGGTTGGACTTGAG | 0.4754 |
| W3 | **SYMME_00004807_79507** | ACGTTGGATGCAAGCCGAAGGCTTGAATTG | ACGTTGGATGAGCTGATTGGCAGGTTGTTC | cccATTTTATTGCTGCACGTA | 0.4442 |
| W3 | **SYMME_00001692_108228** | ACGTTGGATGTTTGGGTCAAACTGGTCGAG | ACGTTGGATGAAGTGTGTAAGTGCACAGAG | ACTGGTCGAGTATTTTGCACA | 0.499 |
| W3 | **SYMME_00001977_272813** | ACGTTGGATGAAGCAGGAGATCATGACCAG | ACGTTGGATGTAGCTTCTTTCAGCCTCCAG | gaGGCAGTACAAAAAGGAGGC | 0.4525 |
| W3 | SYMME_00023242_770809 | ACGTTGGATGGCCAAACGAATCTCAAAGTG | ACGTTGGATGGTTGTAACTTCTCATTGCACG | TCGAACTAGACCTTCACACTTA | 0.5651 |
| W3 | **SYMME_00024209_145735** | ACGTTGGATGACAACAAGACGAGATGCGTG | ACGTTGGATGTCTCGGACCGTGATGAACTG | aACGAGATGCGTGCGATTAATT | 0.4813 |
| W3 | **SYMME_00003452_457206** | ACGTTGGATGTAGCTGTGATACAGCATTAC | ACGTTGGATGAAACAACACGTCACGAGCAC | cTAATCCATGAGATTCTTTCAGG | 0.4452 |
| W3 | SYMME_00024389_110000 | ACGTTGGATGAGGAAGACTCCCACTTTGCC | ACGTTGGATGTGCCTGTGGCTGAAGAACG | gGAGGCAGACGGGTCGAGTCCAG | 0.4154 |
| W3 | SYMME_00001278_696676 | ACGTTGGATGGTCAATTATTGTCTTTCTGC | ACGTTGGATGACAGTGACAGTTTGTAGCTC | cccTCTTTCTGCATATTTTTCGTG | 0.6951 |
| W3 | **SYMME_00000053_1922641** | ACGTTGGATGTGGACATGAAGGAGTCACAC | ACGTTGGATGATATCCCTCCGCTTGCAAAC | accAAAGAAAGGCCCACTAATCTT | 0.5578 |
| W3 | SYMME_00002916_300935 | ACGTTGGATGTTCTGAAGATGACACGACGC | ACGTTGGATGTTAAGAATGCTGCGAACGAG | gaACACTGTTTGCAGGTTACTCGG | 0.4178 |
| W3 | **SYMME_00000230_2293915** | ACGTTGGATGGCTTTCCACTTACAGATACC | ACGTTGGATGGAATATCCCATCCAACCACG | ACAGATACCTAAATGATCTGTACAC | 0.6058 |
| W3 | **SYMME_00023188_229499** | ACGTTGGATGATCGCGACGTGAAATGATGC | ACGTTGGATGTATTATCTGCCGTTAGCGCC | aGCGACGTGAAATGATGCGACTGAT | 0.481 |
| W3 | **SYMME_00002548_480157** | ACGTTGGATGGTACTCGTCTATGAGTCTAC | ACGTTGGATGAGCCATTAACATGCTAACAG | cccTTGACATTCTTAACAGCGATCAC | 0.4602 |
| W3 | **SYMME_00008867_17068** | ACGTTGGATGTCCTGCCAGCGATTGCAATG | ACGTTGGATGGACTTTGAAGGAGCTTGGTG | gCCTGCCAGCGATTGCAATGCAACAA | 0.4949 |
| W3 | **SYMME_00010761_23994** | ACGTTGGATGTGAGTATACCAGAGGAGAGG | ACGTTGGATGCAGCTCAACCGATGCATGTG | cAGAAAAACGGCTGCAGCTTCCAGTT | 0.453 |
| W3 | SYMME_00023181_495503 | ACGTTGGATGGCCGGATGGAGAAACAGCAA | ACGTTGGATGTTAACCGTCAATGGGCCTTG | aCCGGATGGAGAAACAGCAACGAACA | 0.417 |
| W4 | **SYMME_00023385_679926** | ACGTTGGATGTGCTCTTCCTCTCCATTCAC | ACGTTGGATGATAGCCCCAGAATTCACAGC | TCTCCATTCACTGCATG | 0.4402 |
| W4 | **SYMME_00002806_257690** | ACGTTGGATGAAATCCCCCACAAAACCCTG | ACGTTGGATGGCGCAGAGTTATGATACGTG | CCCTGCAGAGGACGAAC | 0.409 |
| W4 | **SYMME_00003210_166008** | ACGTTGGATGCAGAGACTATGTCCTACCTG | ACGTTGGATGTGTCAGTGCTTTTTCTGTGG | CACTGGGCAGCAGCGAT | 0.5152 |
| W4 | **SYMME_00002054_508210** | ACGTTGGATGAAAATGTCTGCATTTTCCGC | ACGTTGGATGGCACATTTTGCTTGGTGAGG | TGCATTTTCCGCATCACT | 0.5552 |
| W4 | **SYMME_00009579_72077** | ACGTTGGATGATTATGGAAGGGAAGCGGAC | ACGTTGGATGTTGGTGCTTTAGACAGACGC | GTGAGGGGACGTTCACTG | 0.4081 |
| W4 | **SYMME_00004127_40394** | ACGTTGGATGAGTGTACAGGCCTGAAAGAC | ACGTTGGATGCTTAAAGGAAACTCGGCGTG | ccTGCCAAATAAACCCGAG | 0.5261 |
| W4 | **SYMME_00024054_274460** | ACGTTGGATGGAAGGCACCTACACTAAGAG | ACGTTGGATGGAACGCTGACCTTGACTTTG | ACTAAGAGTAACGAGCTCC | 0.5084 |
| W4 | **SYMME_00003115_60176** | ACGTTGGATGGGCAATATTCAATAGTGTGTG | ACGTTGGATGCTCTTCCTCTCCATGTTTGC | ATAGTGTGTGTGAGGAAGT | 0.4106 |
| W4 | **SYMME_00011044_1109** | ACGTTGGATGATTCAGTGTCTTCACCGTTG | ACGTTGGATGATATTCAGTCCCTCCTGAGC | GACGATGTGTCTGCAGAGTC | 0.4144 |
| W4 | **SYMME_00000436_144204** | ACGTTGGATGCACAGACTTAGATTTAAGGC | ACGTTGGATGAATTGGTTCAGTGACATTGG | cCTTAGATTTAAGGCAACTCC | 0.4164 |
| W4 | **SYMME_00023191_105880** | ACGTTGGATGTAATGTCCTCTTTCAGGACG | ACGTTGGATGGACAGATTCATGGACCTGAG | CTTTCAGGACGTAATAGACGA | 0.4162 |
| W4 | **SYMME_00024072_36827** | ACGTTGGATGGGAAGATGGATGACAGAGAG | ACGTTGGATGCTGGTGTCAGTCAGGTCTTT | ggcTGACAGAGAGACGAGAGA | 0.4352 |
| W4 | SYMME_00000367_402845 | ACGTTGGATGGTGAGAAGATAACAGACAGG | ACGTTGGATGGTGCTGTCGATTAAATGCTG | cccAACTGCAAATATTTCGAGA | 0.5143 |
| W4 | **SYMME_00006358_220367** | ACGTTGGATGTCACTCTCTACGTAAACCCC | ACGTTGGATGAGCCACAGCTCGAATTGAAC | gAGAATGGGCTTCAGAAGTCAT | 0.5661 |
| W4 | **SYMME_00000564_900003** | ACGTTGGATGTTTGTAACCACAGACTGGGC | ACGTTGGATGATCTGAAACACCGCCTTCAC | tACCGGGTTTCTTCTATAGCTGT | 0.4662 |
| W4 | SYMME_00002174_10525 | ACGTTGGATGAAGCATTCCTGCTGCAAGAC | ACGTTGGATGATCATGTGTACGGCACGTTG | tgaAAGACCAGAGACGATAAGGT | 0.6857 |
| W4 | **SYMME_00023722_192560** | ACGTTGGATGGTGTCATTCTTCGTCTGTCC | ACGTTGGATGGATTAATGACTCCTTGGCCC | ccTCTGTCCTTCAGTAACAAGCAA | 0.616 |
| W4 | SYMME_00003544_104554 | ACGTTGGATGGTGGTTTCAAGGCGCATCAG | ACGTTGGATGATCATGAGGGTAACGTCAGC | tCATCAGGCTCAGCAGGCTGGTCG | 0.5449 |
| W4 | SYMME_00001033_46636 | ACGTTGGATGTGGGTTTGTGCAAGTACTGG | ACGTTGGATGTGATATTTTGAACATATCAC | ggcATGAGGAATGGTCCGATACAT | 0.456 |
| W4 | **SYMME_00023145_4178055** | ACGTTGGATGGGGAAGAAACTCTTCCACAC | ACGTTGGATGACACCAGTGTTGTCAGCTTC | TTCCACACGAGTTAATGTCCAATCA | 0.5307 |
| W4 | SYMME_00000564_674855 | ACGTTGGATGTGGCGTTTTTTGCTGGTCTC | ACGTTGGATGTACACAGAAGCAAAGTGCCG | gCTTTATTTGTGACCCTGTCGGGAA | 0.7344 |
| W4 | **SYMME_00005187_163571** | ACGTTGGATGTAAAGGCACTGCTGTTCAAG | ACGTTGGATGTCTGAATGCAGCAGGCTTAC | aaTGTAGTGTGAAAAAAAAGGGCAA | 0.4056 |
| W4 | **SYMME_00023225_397158** | ACGTTGGATGTCGTGTTTATTCACGGCGTC | ACGTTGGATGCTTTAGTGAGCAGGACCATC | tCTGACACGGTGTCACATTCTCAAAA | 0.4506 |
| W4 | SYMME_00001635_333554 | ACGTTGGATGTACACACGTGTATGCCACTG | ACGTTGGATGAGGAGCTTTGAGAGCAAGTC | gCGTGTATGCCACTGCAGGCTGCGAT | 0.7632 |
| W4 | SYMME_00001467_618978 | ACGTTGGATGGCCTGCACCATTTACACTTG | ACGTTGGATGTGCCAAATTACACACTCTGC | cctACTTTCCTAATTTCTTCAGTAATT | 0.4907 |

Table S2. Number of genotypes, allelic richness (calculated using rarefaction), mean values of unbiased expected heterozygosity, observed heterozygosity, F_IS_ and p-values for global Hardy-Weinberg exact test averaged across 84 loci. STO17 was not included because only one sample was collected there.

| **Geographic Group** | **Sample location** | **Abbreviation** | **N** | **AR** | **Hobs** | **Hexp** | **FIS** | **HWE** |
| --- | --- | --- | --- | --- | --- | --- | --- | --- |
| Mid-West | Flatanger | FLA16 | 95 | 1.824 | 0.376 | 0.396 | 0.055 | 0.001 |
| Mid-West | Flatanger | FLA17 | 307 | 1.849 | 0.396 | 0.413 | 0.045 | 0 |
| Mid-West | Flatanger | FLA18 | 30 | 1.823 | 0.385 | 0.397 | 0.02 | 0.724 |
| Mid-West | Hitra | HIT17 | 10 | 1.797 | 0.382 | 0.382 | -0.064 | 1 |
| Mid-West | Edøya/Smøla | SMO17 | 13 | 1.804 | 0.388 | 0.388 | -0.034 | 0.994 |
| Mid-West | Smøla | SMO18 | 245 | 1.797 | 0.37 | 0.383 | 0.031 | 0 |
| Mid-West | Tustna | TUS17 | 3 | 1.792 | 0.381 | 0.384 | -0.181 | 1 |
| Mid-West | Kristiansund | KRI17 | 43 | 1.792 | 0.367 | 0.381 | 0.021 | 0.019 |
| Mid-West | Averøy | AVE17 | 3 | 1.793 | 0.391 | 0.386 | -0.214 | 1 |
| Mid-West | Sandøy | SAN17 | 3 | 1.813 | 0.367 | 0.379 | -0.173 | 1 |
| Mid-West | Midsund | MID17 | 21 | 1.775 | 0.351 | 0.372 | 0.017 | 0.996 |
| Mid-West | Ålesund | ALE17 | 38 | 1.787 | 0.375 | 0.378 | 0.003 | 0.099 |
| Mid-West | Sula | SUL13 | 77 | 1.785 | 0.354 | 0.378 | 0.06 | 0 |
| Mid-West | Overall Mid-West |  | 888 | 2 | 0.38 | 0.4 | 0.0444 | 0 |
| South-West | Måløy | MAL13 | 5 | 1.817 | 0.36 | 0.396 | -0.039 | 1 |
| South-West | Flora | FLO18 | 9 | 1.825 | 0.353 | 0.398 | 0.072 | 0.999 |
| South-West | Os | OS14 | 134 | 1.822 | 0.405 | 0.398 | -0.012 | 0 |
| South-West | Austevoll | AUS14 | 91 | 1.818 | 0.391 | 0.396 | 0.017 | 0 |
| South-West | Austevoll | AUS17 | 233 | 1.822 | 0.379 | 0.4 | 0.054 | 0 |
| South-West | Sveio | SVE14 | 148 | 1.824 | 0.394 | 0.4 | 0.018 | 0 |
| South-West | Overall South-West | | 620 | 2 | 0.39 | 0.4 | 0.0255 | 0.017 |
| South-East | Årdalsfjorden | ARD18 | 10 | 1.895 | 0.411 | 0.443 | 0.034 | 1 |
| South-East | Flødevigen | FLOD17 | 106 | 1.433 | 0.184 | 0.191 | 0.022 | 1 |
| South-East | Risør | RIS16 | 41 | 1.423 | 0.186 | 0.189 | 0.005 | 1 |
| South-East | Hvaler | HVA14 | 60 | 1.427 | 0.185 | 0.189 | 0.019 | 1 |
| South-East | Marstrand | MAR16 | 40 | 1.428 | 0.187 | 0.189 | -0.014 | 1 |
| South-East | Overall South-East |  | 257 | 1.89 | 0.18 | 0.19 | 0.0255 | 0.928 |

Table S3. Pairwise *F_ST_* between sampling locations. Lower left trimatrix display W&C *F_ST_* estimates and upper right trimatrix display respective p-values. Bold cells have a p-value < 0.05 and grey are significant values after Bonferroni correction

.

Table S4. Model-fitting for the different markers and parameter estimates for the geographic cline ranging from Flatanger to Marstrand. For the given models p_min_ and p_max_ were fixed to 0 and 1 (typ models), or to their empirical values (fix models), or p_min_ and p_max_ are fitted (opt model). Tail fitting encompassed right (R), left (L), none (N) or both fitted (B). The cline width (w) was calculated as 1/maximum slope. Two log-likelihood unit support limits are presented in parentheses for centre and width. Δ and τ are the shape parameters for the left and right tails, and p_min_ and p_max_ are the character states at either end of the transect.

| **Marker** | **Model** | **AICc** | **Centre (km)** | **Width (km)** | **δ_M_** | **τ_M_** | **δ_L_** | **τ_L_** | **p_min_** | **p_max_** | **loglike** |
| --- | --- | --- | --- | --- | --- | --- | --- | --- | --- | --- | --- |
| STRUCTURE Q-score | optN | 627.292 | 799.4 (786.7, 1087.2) | 23.3 (0.0, 168.9) |  |  |  |  | 0.00409 | 0.89123 | -309.6292 |
| SYMME_00000230_2293915 | fixL | 80.234 | 941.1 (768.6, 1044.9) | 224.7 (93.0, 541.4) |  |  | 49.40234 | 0.00004 | 0.56500 | 1.00000 | -36.09707 |
| SYMME_00000351_687296 | fixL | 137.388 | 817.9 (770.7, 1052.2) | 544.2 (149.3, 831.3) |  |  | 94.14454 | 0.00001 | 0.45900 | 0.96700 | -64.67724 |
| SYMME_00001019_259056 | fixL | 116.056 | 767.9 (736.7, 1058.6) | 152.8 (13.7, 362.0) |  |  | 36.67244 | 0.00011 | 0.50000 | 1.00000 | -54.01118 |
| SYMME_00001278_977669 | fixL | 126.596 | 884.6 (832.1, 1056.2) | 384.1 (100.0, 529.2) |  |  | 146.75380 | 0.00011 | 0.44800 | 0.98800 | -59.28127 |
| SYMME_00001692_108228 | fixL | 203.006 | 927.6 (843.2, 1064.3) | 448.2 (83.6, 601.8) |  |  | 210.63860 | 0.00005 | 0.37500 | 1.00000 | -97.48641 |
| SYMME_00001977_272813 | fixL | 125.701 | 818.3 (854.0, 1065.1) | 674.6 (81.5, 535.7) |  |  | 86.70604 | 0.00024 | 0.00000 | 1.00000 | -58.822 |
| SYMME_00002548_480157 | fixL | 177.925 | 875.6 (773.8, 1080.6) | 312.2 (22.0, 530.8) |  |  | 118.25190 | 0.00005 | 0.15700 | 0.84200 | -84.94583 |
| SYMME_00002806_257690 | fixL | 68.882 | 912.8 (757.4, 1077.1) | 420.2 (30.2, 717.0) |  |  | 151.04970 | 0.00002 | 0.47300 | 0.86800 | -30.42424 |
| SYMME_00002850_26023 | fixL | 324.532 | 810.8 (762.7, 1048.8) | 331.5 (48.0, 430.7) |  |  | 92.20118 | 0.00005 | 0.01300 | 0.97600 | -158.2491 |
| SYMME_00003452_457206 | fixL | 129.891 | 814.7 (752.9, 1050.1) | 161.4 (5.7, 334.7) |  |  | 54.49966 | 0.00014 | 0.43900 | 1.00000 | -60.92881 |
| SYMME_00005187_163571 | fixL | 141.773 | 883.9 (776.5, 1066.6) | 474.6 (45.4, 669.6) |  |  | 169.39900 | 0.00031 | 0.37500 | 0.95000 | -66.86962 |
| SYMME_00006969_69288 | fixL | 226.462 | 906.1 (825.2, 1058.4) | 445.8 (83.1, 672.5) |  |  | 136.31460 | 0.00013 | 0.12700 | 0.92100 | -109.2142 |
| SYMME_00009579_72077 | fixL | 83.769 | 818.5 (747.4, 1061.4) | 364.5 (23.1, 604.8) |  |  | 88.22731 | 0.00003 | 0.05000 | 0.59500 | -37.86781 |
| SYMME_00013619_22969 | fixL | 112.525 | 757.2 (732.2, 1049.1) | 285.4 (47.1, 463.1) |  |  | 35.72526 | 0.00001 | 0.40000 | 1.00000 | -52.24568 |
| SYMME_00023191_105880 | fixL | 125.618 | 778.1 (733.4, 1045.9) | 195.4 (15.3, 419.4) |  |  | 34.41165 | 0.00007 | 0.55900 | 1.00000 | -58.79201 |
| SYMME_00023242_777958 | fixL | 102.751 | 774.8 (725.7, 1037.6) | 161.9 (9.9, 394.7) |  |  | 28.26784 | 0.00010 | 0.40700 | 1.00000 | -47.35829 |
| SYMME_00024072_36827 | fixL | 116.062 | 911.3 (835.6, 1060.8) | 497.8 (78.4, 655.0) |  |  | 192.49440 | 0.00019 | 0.21300 | 0.85000 | -54.01428 |
| SYMME_00000053_1922641 | optN | 72.140 | 810.4 (780.8, 1085.3) | 3.2 (0.0, 227.9) |  |  |  |  | 0.00002 | 0.51431 | -32.04971 |
| SYMME_00000097_411069 | optN | 59.272 | 862.5 (775.2, 1089.4) | 1.4 (0.0, 319.0) |  |  |  |  | 0.79811 | 0.99647 | -25.61927 |
| SYMME_00000109_1247032 | optN | 267.679 | 834.9 (787.0, 1088.0) | 11.4 (0.0, 180.3) |  |  |  |  | 0.00005 | 0.69776 | -129.8228 |
| SYMME_00000375_42132 | optN | 108.158 | 784.9 (723.9, 1086.0) | 17.3 (0.0, 332.7) |  |  |  |  | 0.14571 | 0.65653 | -50.06171 |
| SYMME_00000436_144204 | optN | 95.703 | 799.1 (730.7, 966.2) | 304.3 (117.5, 645.4) |  |  |  |  | 0.06925 | 0.51413 | -43.83465 |
| SYMME_00000564_900003 | optN | 81.569 | 993.3 (786.6, 1088.9) | 82.2 (0.0, 344.7) |  |  |  |  | 0.47619 | 0.88151 | -36.76738 |
| SYMME_00001073_633173 | optN | 72.192 | 706.6 (685.5, 790.0) | 154.8 (25.0, 493.1) |  |  |  |  | 0.75541 | 0.98683 | -32.079 |
| SYMME_00001181_204509 | optN | 97.772 | 786.5 (771.8, 1087.7) | 5.2 (0.0, 249.4) |  |  |  |  | 0.67596 | 0.99681 | -44.8694 |
| SYMME_00001242_101541 | optN | 109.745 | 781.3 (717.2, 1086.8) | 26.6 (0.0, 247.7) |  |  |  |  | 0.61193 | 0.98244 | -50.85559 |
| SYMME_00001599_151719 | optN | 118.294 | 811.2 (777.7, 1088.6) | 38.8 (0.0, 260.5) |  |  |  |  | 0.62308 | 0.99653 | -55.13 |
| SYMME_00001760_736838 | optN | 172.044 | 788.4 (720.9, 1090.2) | 15.2 (0.0, 271.4) |  |  |  |  | 0.45939 | 0.88288 | -82.00522 |
| SYMME_00002054_508210 | optN | 170.245 | 811.3 (782.6, 1088.4) | 41.8 (0.0, 237.5) |  |  |  |  | 0.22122 | 0.78816 | -81.10554 |
| SYMME_00002115_94621 | optN | 137.893 | 803.5 (764.0, 1087.3) | 49.1 (0.0, 317.1) |  |  |  |  | 0.08890 | 0.57843 | -64.92947 |
| SYMME_00002116_350960 | optN | 207.230 | 985.1 (785.4, 1089.8) | 38.2 (0.0, 305.3) |  |  |  |  | 0.12829 | 0.67438 | -99.59824 |
| SYMME_00002315_632632 | optN | 51.997 | 791.0 (729.4, 1175.8) | 10.7 (0.1, 623.2) |  |  |  |  | 0.36571 | 0.75265 | -21.97763 |
| SYMME_00002662_67836 | optN | 144.101 | 1056.2 (785.9, 1105.3) | 90.6 (0.0, 317.6) |  |  |  |  | 0.12573 | 0.61083 | -68.03384 |
| SYMME_00002972_463186 | optN | 122.806 | 850.6 (753.0, 1090.4) | 186.2 (0.0, 540.4) |  |  |  |  | 0.17075 | 0.57603 | -57.38504 |
| SYMME_00003115_60176 | optN | 100.931 | 800.1 (773.0, 1087.6) | 6.8 (0.0, 401.7) |  |  |  |  | 0.28786 | 0.70577 | -46.44891 |
| SYMME_00003129_299972 | optN | 211.385 | 791.9 (775.2, 1088.7) | 27.9 (0.0, 217.6) |  |  |  |  | 0.07304 | 0.64361 | -101.6756 |
| SYMME_00003318_375819 | optN | 174.911 | 787.6 (786.0, 1088.6) | 3.6 (0.0, 244.3) |  |  |  |  | 0.08229 | 0.67692 | -83.43841 |
| SYMME_00003351_50140 | optN | 328.985 | 1036.3 (786.4, 1090.5) | 57.6 (0.0, 223.0) |  |  |  |  | 0.06836 | 0.81013 | -160.4756 |
| SYMME_00003452_329369 | optN | 126.138 | 957.6 (781.9, 1089.3) | 53.4 (0.0, 218.0) |  |  |  |  | 0.58967 | 0.99997 | -59.0514 |
| SYMME_00004127_40394 | optN | 211.269 | 954.9 (785.2, 1088.9) | 249.5 (0.0, 502.2) |  |  |  |  | 0.11255 | 0.69296 | -101.6174 |
| SYMME_00004729_285551 | optN | 276.066 | 799.3 (782.7, 1090.5) | 30.7 (0.0, 207.2) |  |  |  |  | 0.02135 | 0.74185 | -134.0164 |
| SYMME_00004764_285875 | optN | 169.743 | 785.8 (724.5, 1089.4) | 13.6 (0.0, 332.7) |  |  |  |  | 0.26258 | 0.74755 | -80.85494 |
| SYMME_00004807_79507 | optN | 100.326 | 779.8 (722.5, 1089.2) | 44.1 (0.1, 351.6) |  |  |  |  | 0.56357 | 0.96702 | -46.14467 |
| SYMME_00005187_201142 | optN | 192.819 | 838.3 (771.9, 1070.2) | 304.9 (2.0, 423.4) |  |  |  |  | 0.00029 | 0.52992 | -92.39211 |
| SYMME_00006358_220367 | optN | 214.920 | 807.9 (780.5, 1088.0) | 32.2 (0.0, 279.7) |  |  |  |  | 0.30455 | 0.86892 | -103.4423 |
| SYMME_00006679_62104 | optN | 191.850 | 895.4 (782.5, 1087.8) | 15.4 (0.0, 212.7) |  |  |  |  | 0.00003 | 0.53221 | -91.90804 |
| SYMME_00007078_108905 | optN | 204.570 | 806.0 (776.1, 1090.7) | 57.8 (0.0, 275.3) |  |  |  |  | 0.12339 | 0.70402 | -98.26834 |
| SYMME_00007129_47437 | optN | 134.095 | 741.4 (716.5, 810.2) | 2.9 (0.0, 107.9) |  |  |  |  | 0.20898 | 0.70077 | -63.03017 |
| SYMME_00007721_148340 | optN | 208.301 | 787.7 (717.8, 1084.4) | 32.3 (0.1, 182.7) |  |  |  |  | 0.32865 | 0.85839 | -100.1331 |
| SYMME_00008787_21327 | optN | 207.892 | 788.2 (776.6, 1086.5) | 12.2 (0.0, 265.9) |  |  |  |  | 0.07351 | 0.62437 | -99.92902 |
| SYMME_00008867_17068 | optN | 142.177 | 806.4 (774.7, 1087.7) | 29.0 (0.0, 224.9) |  |  |  |  | 0.57553 | 0.99992 | -67.07149 |
| SYMME_00010158_75346 | optN | 105.307 | 857.8 (720.0, 1153.1) | 327.8 (0.2, 836.1) |  |  |  |  | 0.26277 | 0.67423 | -48.63661 |
| SYMME_00010876_30588 | optN | 121.158 | 784.9 (723.8, 1089.5) | 20.0 (0.0, 275.3) |  |  |  |  | 0.52326 | 0.95012 | -56.562 |
| SYMME_00011044_1109 | optN | 149.536 | 875.5 (775.8, 1087.5) | 53.4 (0.0, 382.9) |  |  |  |  | 0.17492 | 0.63277 | -70.75142 |
| SYMME_00011099_16235 | optN | 148.623 | 782.4 (718.0, 870.9) | 29.1 (0.0, 137.7) |  |  |  |  | 0.29368 | 0.76379 | -70.29453 |
| SYMME_00012017_14751 | optN | 139.430 | 1016.2 (779.0, 1087.8) | 9.0 (0.0, 237.1) |  |  |  |  | 0.51388 | 0.98933 | -65.69758 |
| SYMME_00023145_2392136 | optN | 129.217 | 829.9 (757.8, 1089.7) | 202.5 (0.0, 381.0) |  |  |  |  | 0.68376 | 0.99980 | -60.5915 |
| SYMME_00023145_4178055 | optN | 142.693 | 812.3 (771.9, 1087.4) | 49.7 (0.0, 243.8) |  |  |  |  | 0.56730 | 0.97083 | -67.32976 |
| SYMME_00023188_229499 | optN | 132.348 | 1054.5 (784.6, 1156.4) | 99.4 (0.1, 382.2) |  |  |  |  | 0.31053 | 0.76573 | -62.15717 |
| SYMME_00023197_121823 | optN | 121.933 | 784.5 (750.6, 1086.7) | 67.5 (0.0, 246.9) |  |  |  |  | 0.62361 | 0.99994 | -56.94974 |
| SYMME_00023225_397158 | optN | 123.050 | 823.7 (779.4, 1089.8) | 70.1 (0.0, 311.8) |  |  |  |  | 0.13794 | 0.70899 | -57.50637 |
| SYMME_00023225_405765 | optN | 250.733 | 798.5 (783.1, 1089.7) | 16.8 (0.0, 242.3) |  |  |  |  | 0.18547 | 0.82220 | -121.3499 |
| SYMME_00023262_816209 | optN | 128.969 | 786.2 (776.9, 1086.6) | 1.1 (0.0, 268.5) |  |  |  |  | 0.54069 | 0.94564 | -60.46736 |
| SYMME_00023308_56447 | optN | 131.185 | 805.9 (775.0, 1088.6) | 39.8 (0.0, 262.4) |  |  |  |  | 0.05107 | 0.53964 | -61.57515 |
| SYMME_00023319_1170712 | optN | 158.398 | 1062.1 (785.4, 1089.3) | 21.8 (0.0, 277.1) |  |  |  |  | 0.09444 | 0.60554 | -75.18232 |
| SYMME_00023380_3836 | optN | 122.873 | 785.1 (738.9, 1085.6) | 9.2 (0.1, 309.6) |  |  |  |  | 0.11138 | 0.54200 | -57.41965 |
| SYMME_00023385_679926 | optN | 87.845 | 1034.5 (776.4, 1118.0) | 119.3 (0.0, 480.5) |  |  |  |  | 0.15007 | 0.55965 | -39.90547 |
| SYMME_00023453_96630 | optN | 320.818 | 806.8 (783.7, 1090.1) | 38.3 (0.0, 237.0) |  |  |  |  | 0.31280 | 0.90341 | -156.3922 |
| SYMME_00023529_208893 | optN | 99.745 | 790.6 (763.5, 1087.2) | 14.1 (0.0, 293.0) |  |  |  |  | 0.20324 | 0.64053 | -45.85568 |
| SYMME_00023574_386442 | optN | 198.977 | 811.0 (782.9, 1088.8) | 42.6 (0.0, 284.4) |  |  |  |  | 0.31359 | 0.82960 | -95.47196 |
| SYMME_00023722_192560 | optN | 242.737 | 793.6 (785.0, 1088.9) | 21.3 (0.0, 205.6) |  |  |  |  | 0.00388 | 0.63842 | -117.3514 |
| SYMME_00023738_421399 | optN | 159.557 | 1016.7 (785.6, 1087.3) | 81.3 (0.0, 335.3) |  |  |  |  | 0.21937 | 0.74524 | -75.76167 |
| SYMME_00023818_156483 | optN | 90.835 | 965.0 (783.0, 1087.7) | 34.8 (0.1, 238.1) |  |  |  |  | 0.69060 | 0.99999 | -41.40093 |
| SYMME_00023953_207211 | optN | 153.121 | 788.4 (768.2, 1090.0) | 27.2 (0.0, 246.5) |  |  |  |  | 0.53889 | 0.91627 | -72.54345 |
| SYMME_00024054_274460 | optN | 159.753 | 945.7 (786.5, 1085.5) | 9.5 (0.0, 195.3) |  |  |  |  | 0.52945 | 0.99995 | -75.8596 |
| SYMME_00024110_27315 | optN | 74.054 | 866.3 (780.9, 1088.9) | 36.6 (0.0, 361.3) |  |  |  |  | 0.25477 | 0.63648 | -33.00966 |
| SYMME_00024144_124879 | optN | 126.689 | 989.9 (785.1, 1088.4) | 68.6 (0.0, 275.8) |  |  |  |  | 0.50817 | 0.92971 | -59.32702 |
| SYMME_00024209_145735 | optN | 111.076 | 1005.4 (773.1, 1087.4) | 34.4 (0.0, 225.4) |  |  |  |  | 0.70144 | 0.99998 | -51.52109 |
| SYMME_00002674_685013 | typL | 171.673 | 798.0 (723.9, 1012.1) | 366.5 (129.4, 534.9) |  |  | 14.12170 | 0.00002 | 0.00000 | 1.00000 | -81.81944 |
| SYMME_00003210_166008 | typL | 192.638 | 826.0 (770.2, 1014.1) | 466.4 (196.1, 651.6) |  |  | 63.11648 | 0.00001 | 0.00000 | 1.00000 | -92.30223 |
| SYMME_00006025_40517 | typL | 100.855 | 731.0 (698.4, 1015.5) | 718.8 (243.7, 932.8) |  |  | 14.36357 | 0.00005 | 0.00000 | 1.00000 | -46.41094 |
| SYMME_00010761_23994 | typL | 87.767 | 1030.2 (975.0, 1108.9) | 737.5 (356.1, 986.5) |  |  | 278.61970 | 0.00027 | 0.00000 | 1.00000 | -39.8663 |
| SYMME_00001871_333256 | typM | 194.425 | 944.0 (791.9, 1073.6) | 660.2 (33.2, 867.2) | 210.28310 | 0.00006 |  |  | 0.00000 | 1.00000 | -93.19481 |
| SYMME_00024098_237080 | typM | 171.621 | 944.5 (782.6, 1075.8) | 760.8 (57.7, 980.7) | 224.89700 | 0.00005 |  |  | 0.00000 | 1.00000 | -81.79336 |

**Supplementary figures**

Figure S1. Hybrid detection accuracy, efficiency and power at different critical posterior probability thresholds. Solid lines are averages of three replicates of three simulated genotype data sets for 106 SNPs (A-C) and 84 SNPs (D-F). The dashed lines show the standard deviation among the simulations. Colours represent the 6 genotype classes, Pure1 = western population, Pure2 = south-eastern population, F1, F2 BC1 = F1 backcrosses with western population, and BC2 = F1 backcrosses with south-eastern populations. Accuracy = correctly assigned individuals over total individuals assigned to that class. Efficiency = correctly assigned individuals over the known individuals per class. Power = Accuracy * Efficiency. A) Accuracy shows that at critical posterior probability thresholds between 0.5 and 1.0, of the individuals assigned to a given class, > 98% of them will have been assigned correctly. B) Efficiency indicates that > 94% of individuals in each class will be identified at critical posterior probability thresholds between 0.5 and 0.9. C) Which results in power > 0.94 at critical posterior probability threshold between 05 and 0.9 D) Accuracy shows that at critical posterior probability thresholds between 0.5 and 1.0, of the individuals assigned to a given class, > 92% of them will have been assigned correctly. E) Efficiency indicates that > 83% of individuals in each class will be identified at critical posterior probability thresholds between 0.5 and 0.9. F) Which results in power > 0.81 at critical posterior probability threshold between 0.5 and 0.9.


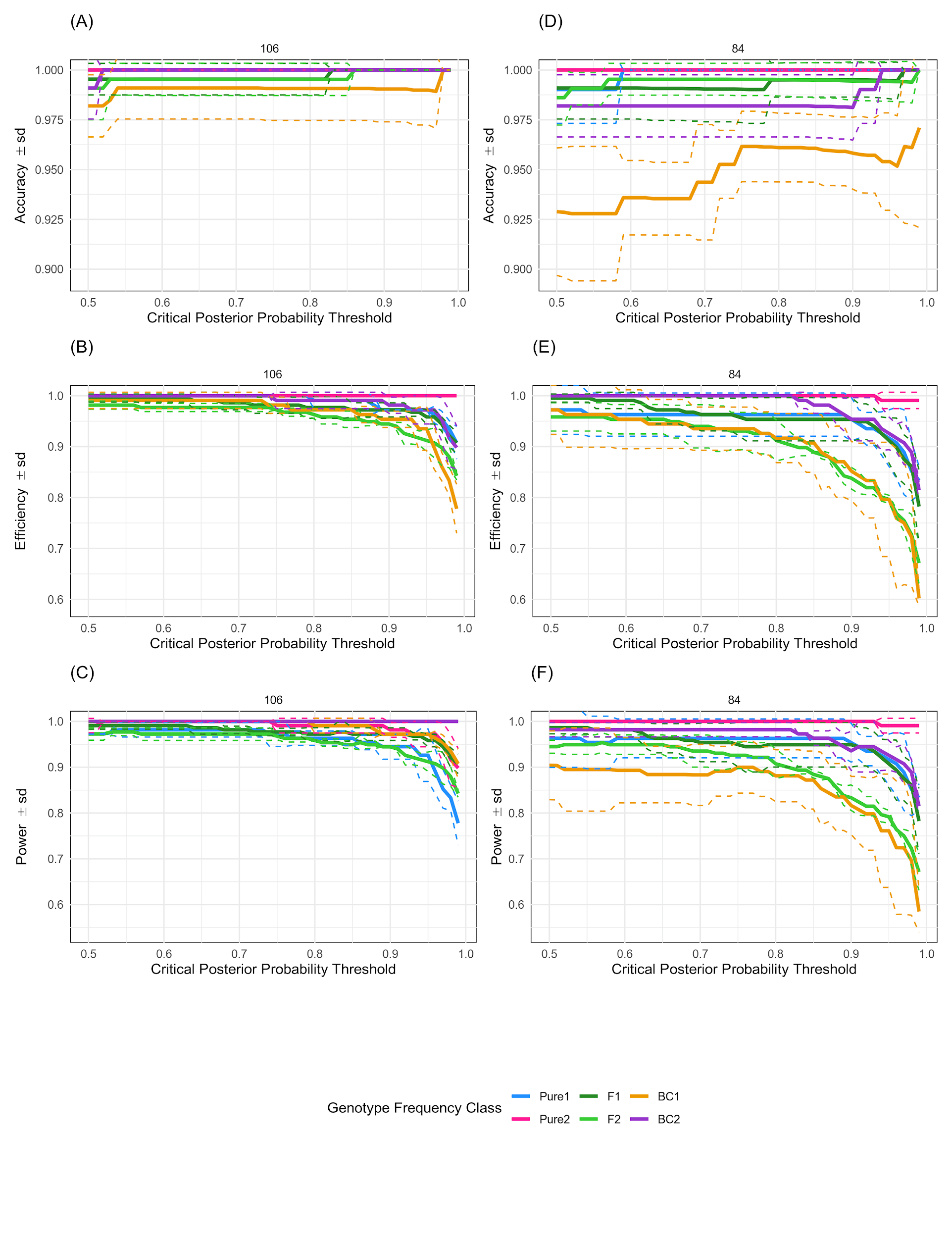


F
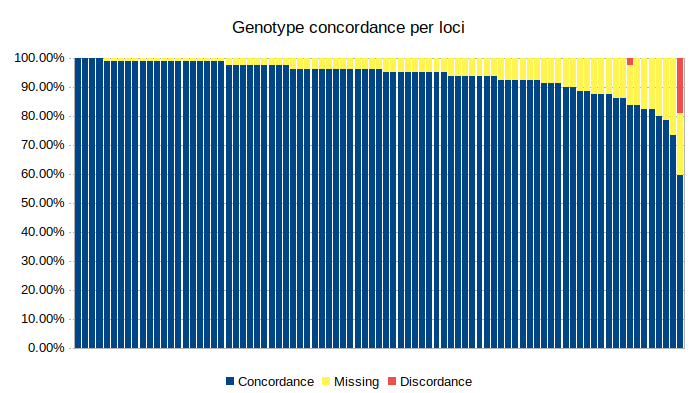
igure S2. Genotyping robustness was evaluated by calculating concordance between 79 successfully genotyped technical replicates across 85 loci. Figure displays A) genotype concordance by locus and B) genotype concordance by individual for 79 technical replicates across 85 loci. Each bar represents the proportion of genotypes that were concordant (i.e. identical), missing (i.e. one or both replicates could not be genotyped) or discordant (i.e.
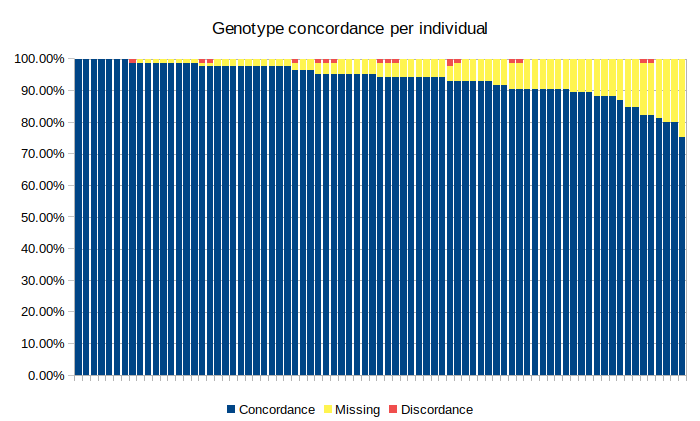
replicates had different genotypes).

A)

B)

Figure S3. Hybrid detection accuracy, efficiency and power at different critical posterior probability thresholds. Solid lines are averages of three replicates of three simulated genotype data sets for 106 SNPs (A-C) and 84 SNPs (D-F). The dashed lines show the standard deviation among the simulations. Colours represent the 3 genotypes, Pure1 = western population, Pure2 = south-eastern population, Hybrid = first or second generation hybrid. Accuracy = correctly assigned individuals over total individuals assigned to that class. Efficiency = correctly assigned individuals over the known individuals per class. Power = Accuracy * Efficiency. A) Accuracy shows that at critical posterior probability thresholds between 0.5 and 1.0, of the individuals assigned to a given class, > 98% of them will have been assigned correctly. B) Efficiency indicates that > 97% of individuals in each class will be identified at critical posterior probability thresholds between 0.5 and 0.9. C) Which results in power > 0.97 at critical posterior probability threshold between 05 and 0.9 D) Accuracy shows that at critical posterior probability thresholds between 0.5 and 1.0, of the individuals assigned to a given class, > 97% of them will have been assigned correctly. E) Efficiency indicates that > 95% of individuals in each class will be identified at critical posterior probability thresholds between 0.5 and 0.9. F) Which results in power > 0.95 at critical posterior probability threshold between 05 and 0.9.


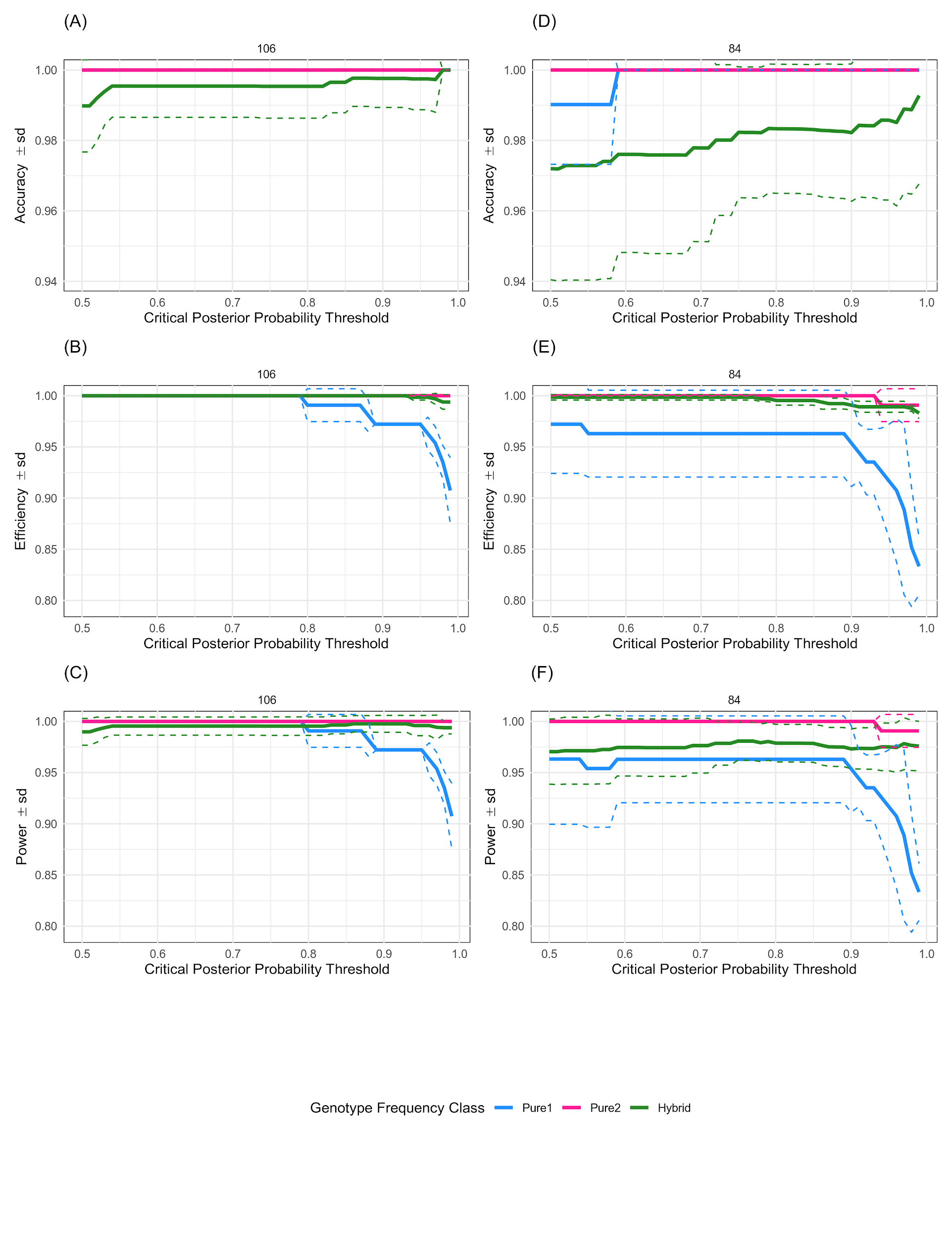


Figure S4. Pairwise estimates for each sample population pair. Upper right: Pearson’s correlation coefficient, lower left: scatter plots of pairwise sample allele frequency, diagonal: density plots of pairwise sample allele frequency. Colors represent alternate alleles. For reference of sample names, see Table 1.


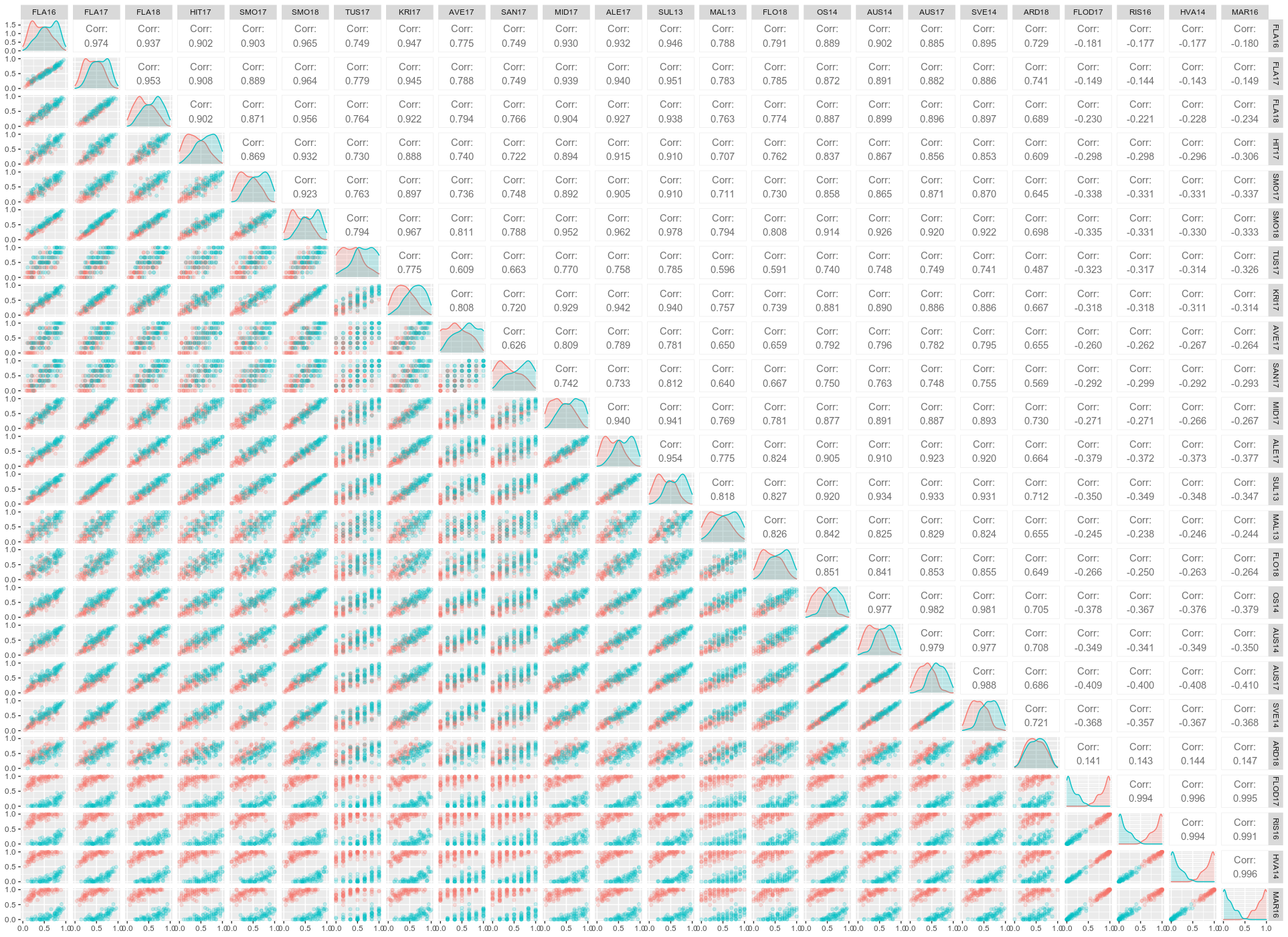


Figure S5a-c. Results from Structure cluster analysis for optimal K using different approaches. Red lines in each figure shows the most supported solution for that particular approach.

S5a. The most likely number of K (5) based on the log probability of data (lnPrX│K).

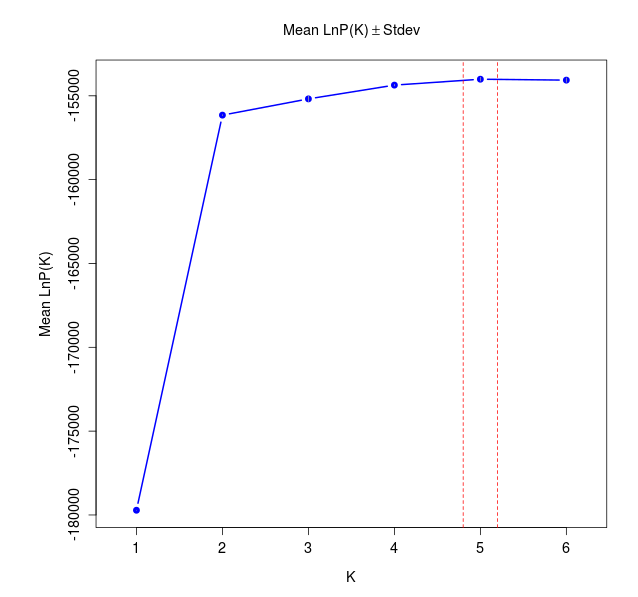


S5b. The most likely number of K (2) based on ΔK.


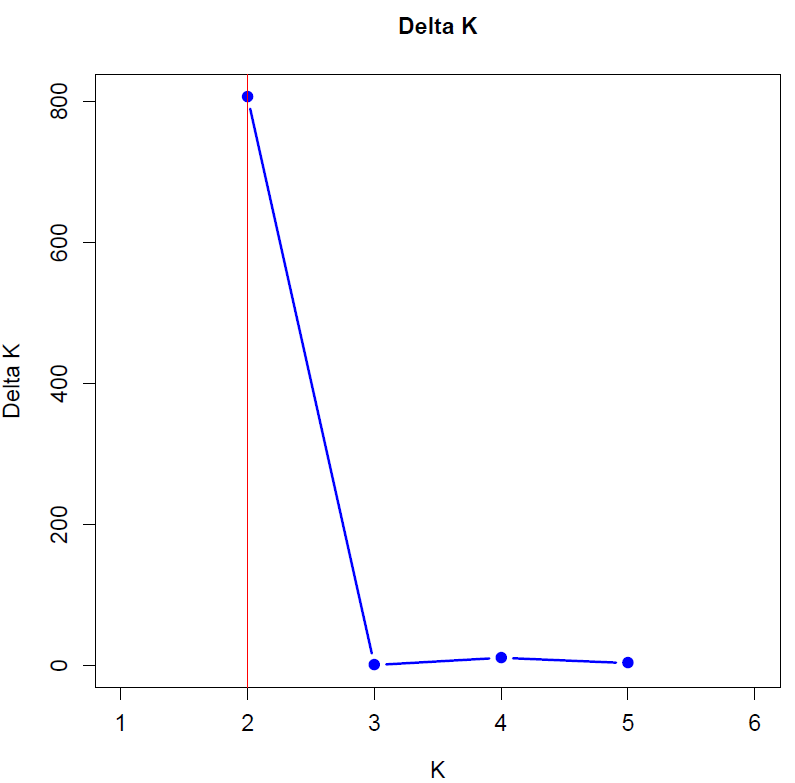


S5c. The most likely number of K (4 or 5) based on four different Puechmaille method calculations.
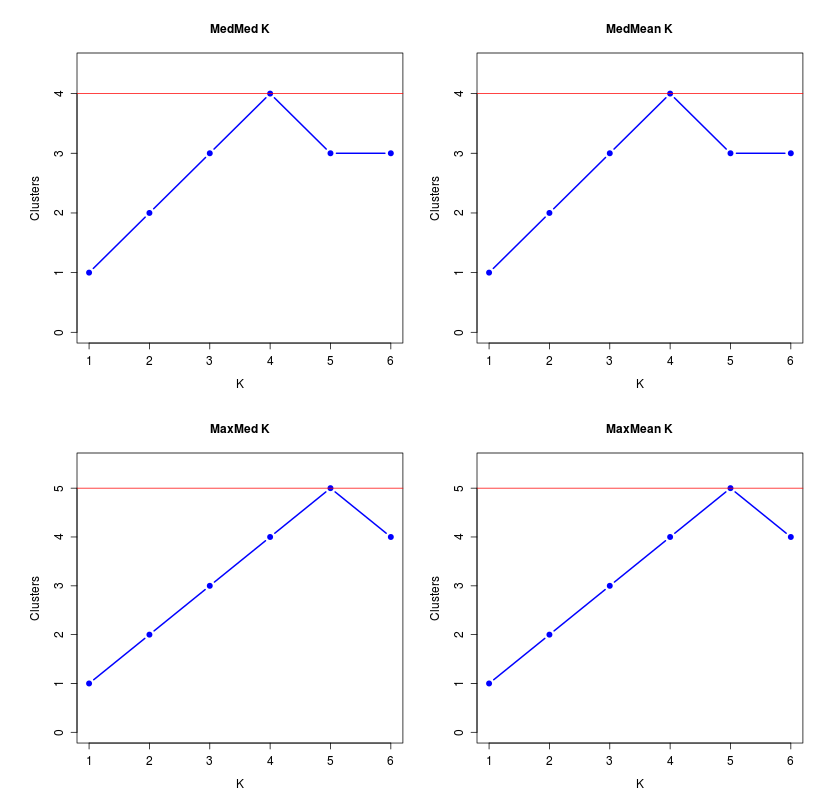


Figure S6a. Structure bar plots for K values from 1 to 6 without a priori.


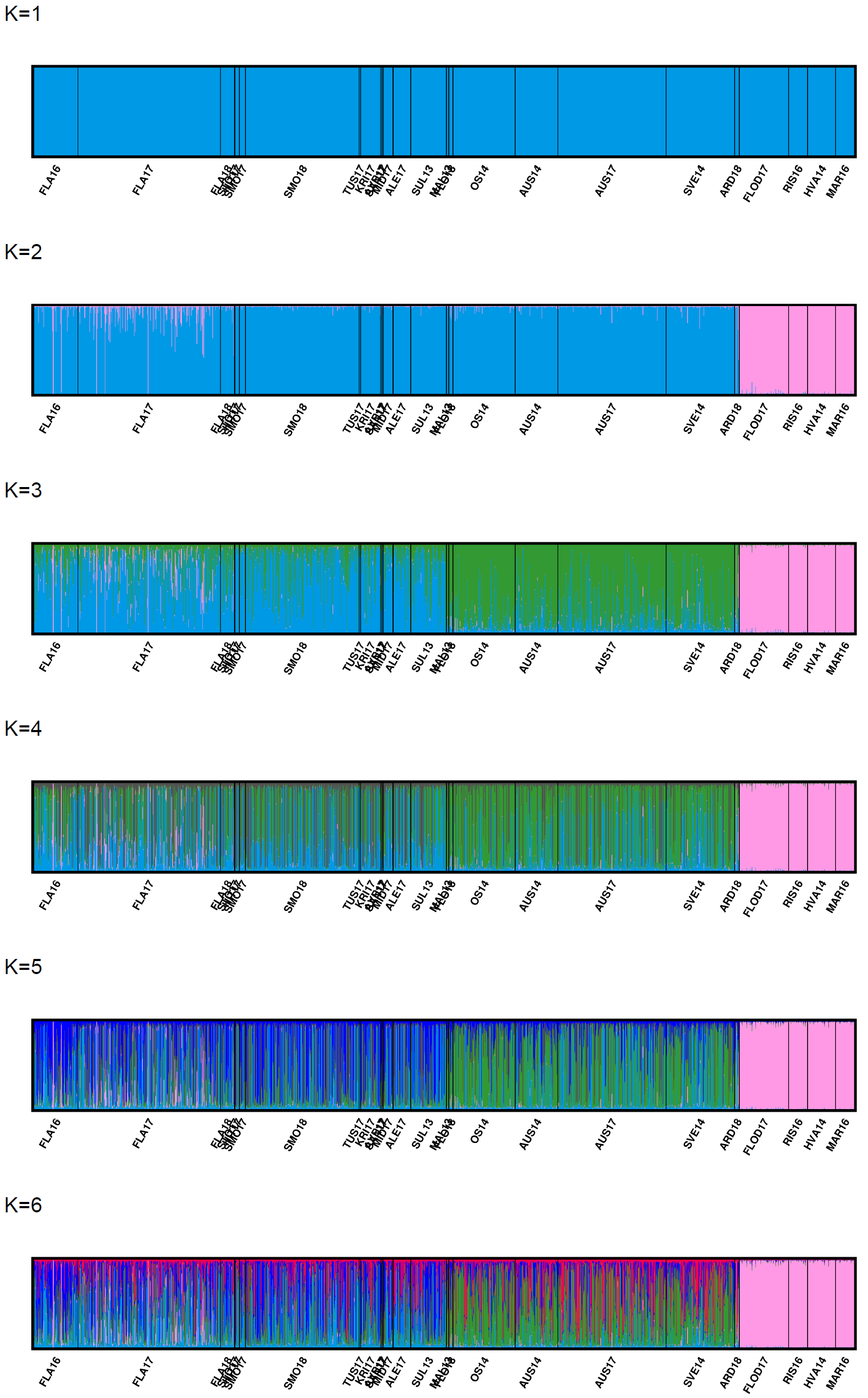


Figure S6b. Structure bar plots for K values from 1 to 6 with sampling location given as a priori.


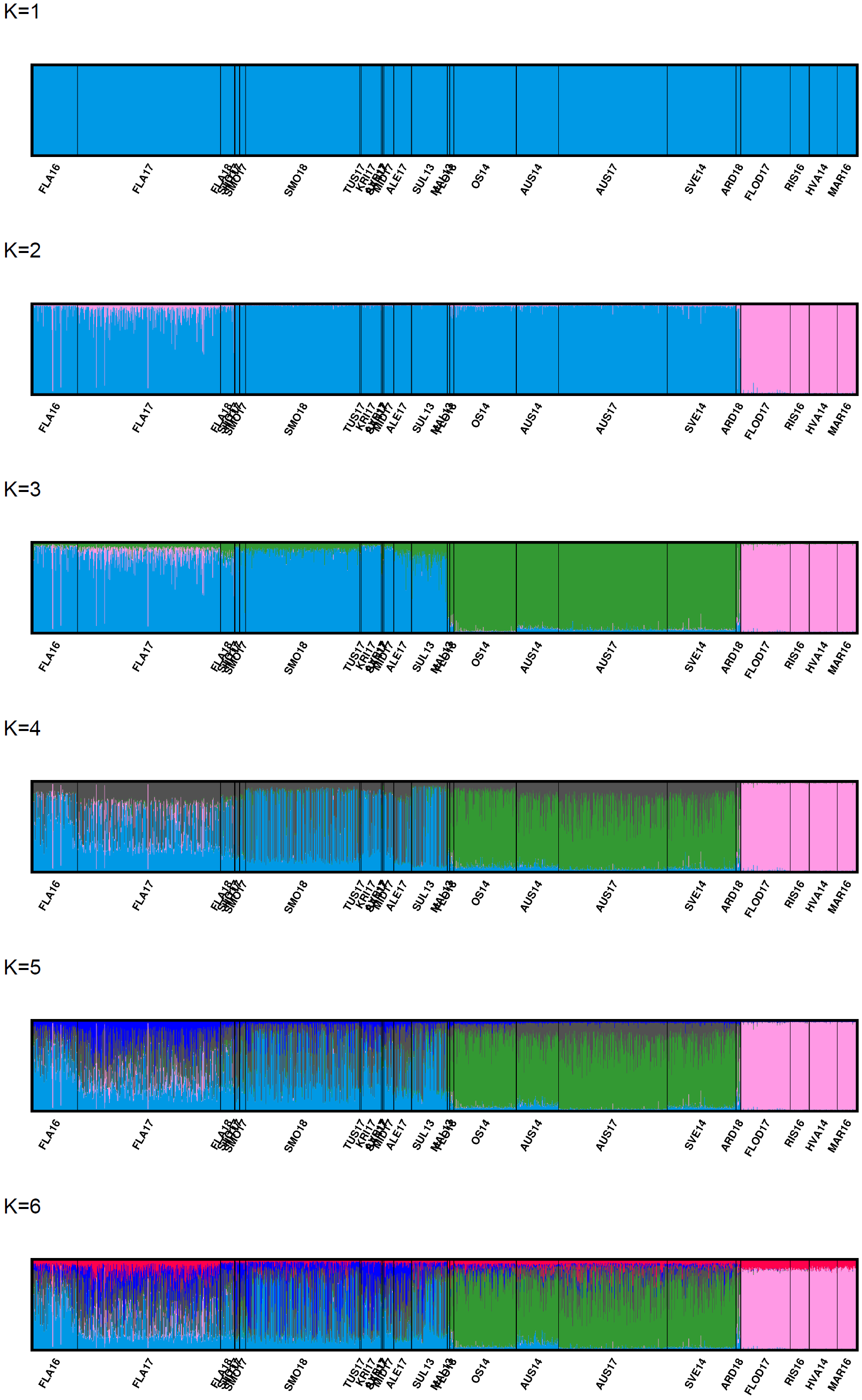


Figure S7. First (x-axis) and second (y-axis) component of a principal component analysis (PCA) on 1766 corkwing wrasse individuals based on 84 SNPs. The first component explains 26.5% of the total variation and the second 2.2%. Each point represents one individual and colours represent the different samples. For reference see Table 1


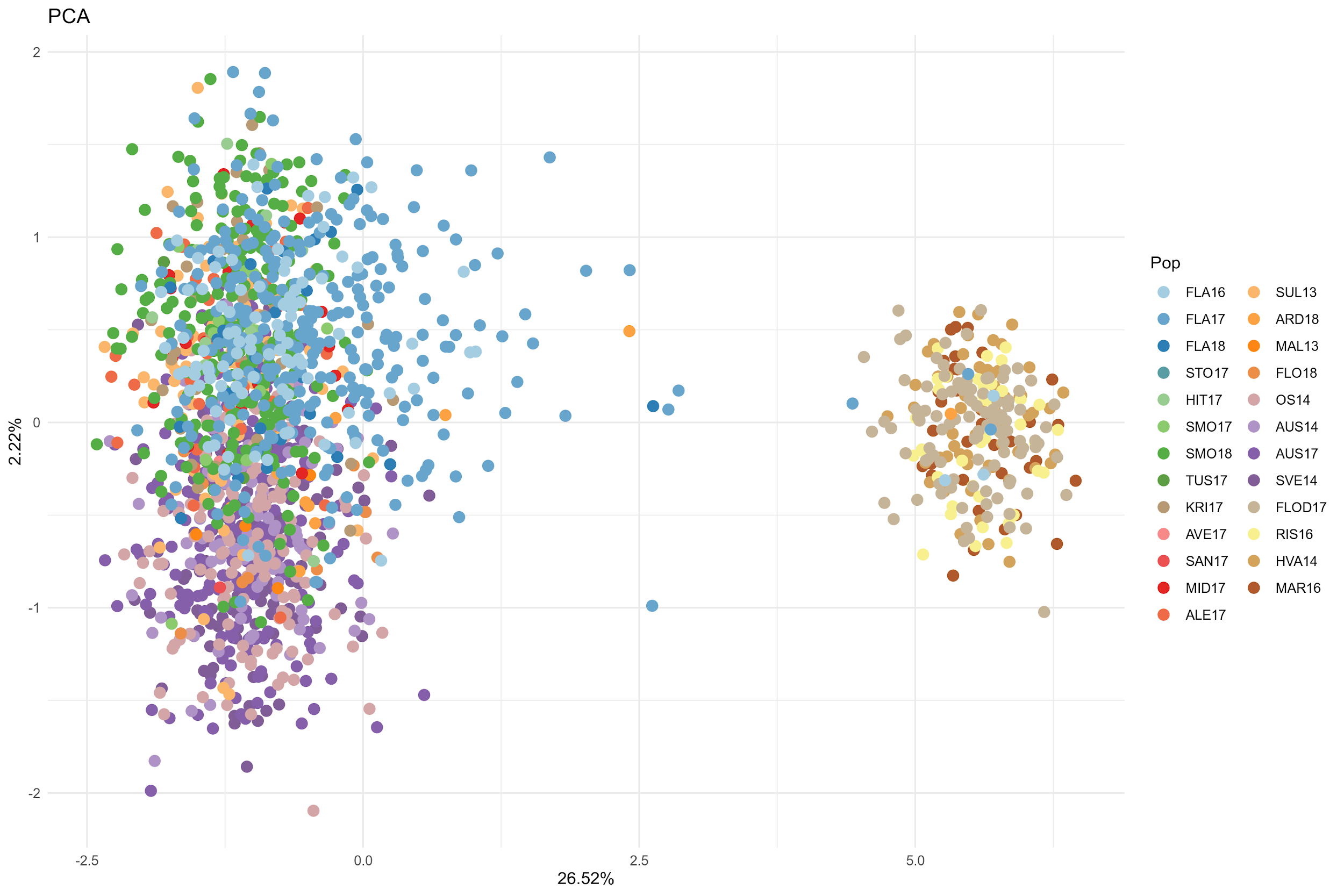


Figure S8a. Treemix analysis. Plotted is a maximum-likelihood tree with no migration events and five trees inferred by TreeMix, allowing for one to five migration events. Migration arrows are colored according to their migration weight and go in the direction from origin to destination. Horizontal branch lengths are proportional to the amount of genetic drift that has occurred on the branch based on allele frequencies. The scale bar shows ten times the averaged standard error of the sample covariance matrix. The residual fit from this graph is shown in Figure S7b. For reference of sample names see Table 1.


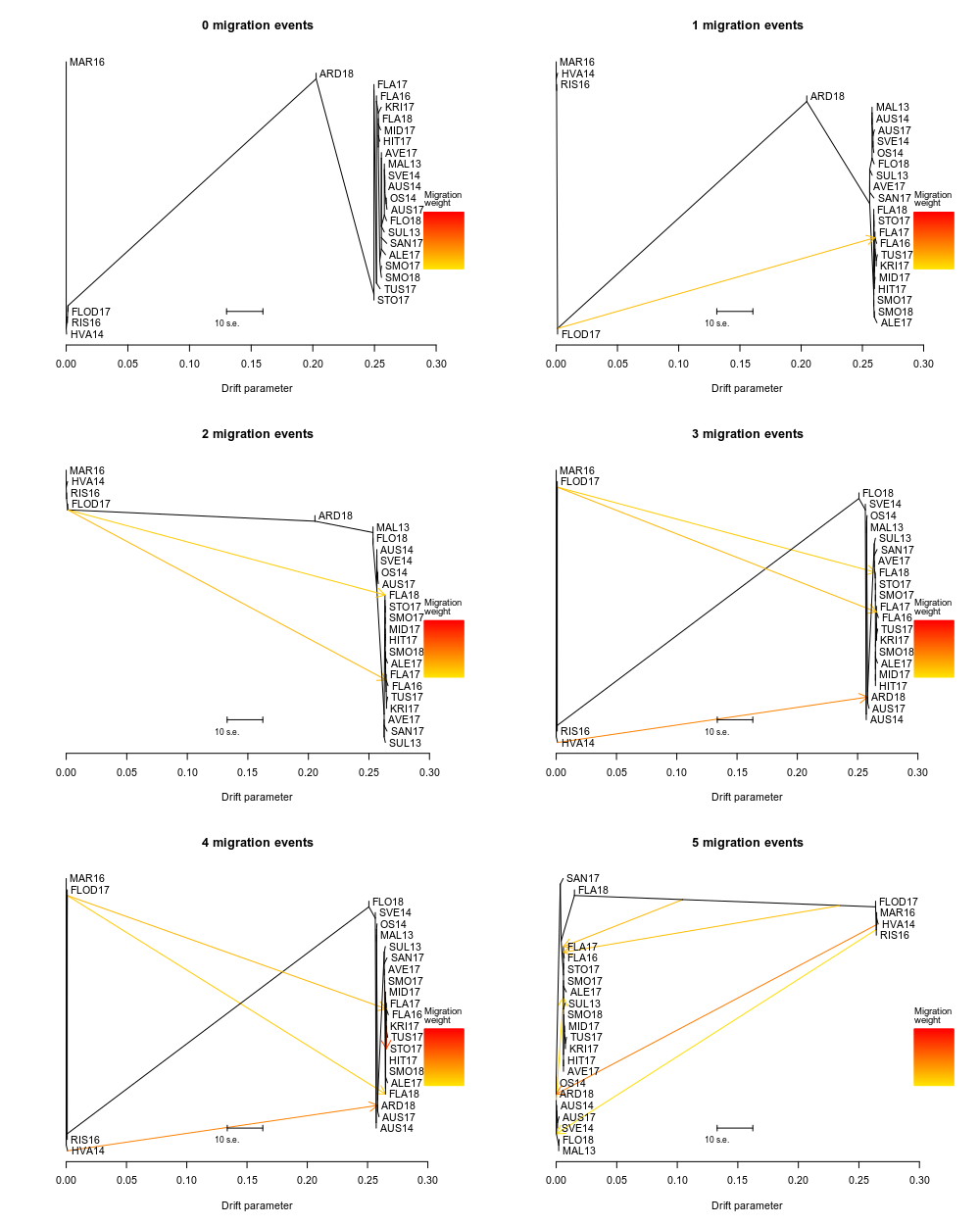


Figure S8b. Plotted is the residual fit of a maximum-likelihood tree with no migration events and five trees inferred by TreeMix, allowing for one to five migration events as seen in Figure S7a. Matrix of residuals indicates how well the tree model fits the data. Positive residuals indicate pairs of populations that are more closely related to each other than in the best-fit tree and are candidates for admixture. Negative residuals indicate pairs of populations where the model overestimates the observed covariance, which is a consequence of having positive residuals, but can also indicate that populations are less related than represented in the best-fit tree. For reference of sample names, see Table 1.


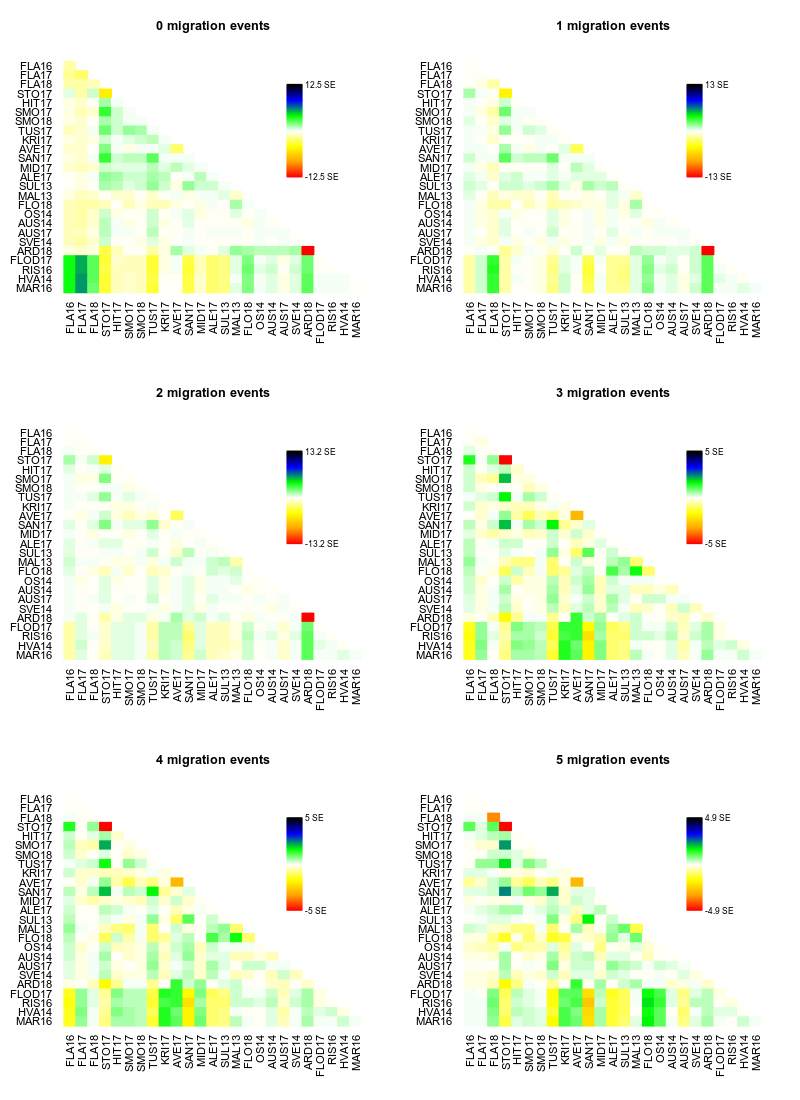


Figure S9. Geographical cline analysis for corkwing wrasse: a) Reference cline based on the STRUCTURE Q-score, and b) cline centres (and their support limits based on two log-likelihood units, in km) obtained by fitting curves for every SNP locus. Cline centres are measured as the distance along the 1200 km long transect ranging from Flatanger to Marstrand. The red dashed lines depict upper and lower values for STRUCTURE Q-score reference cline.

A)

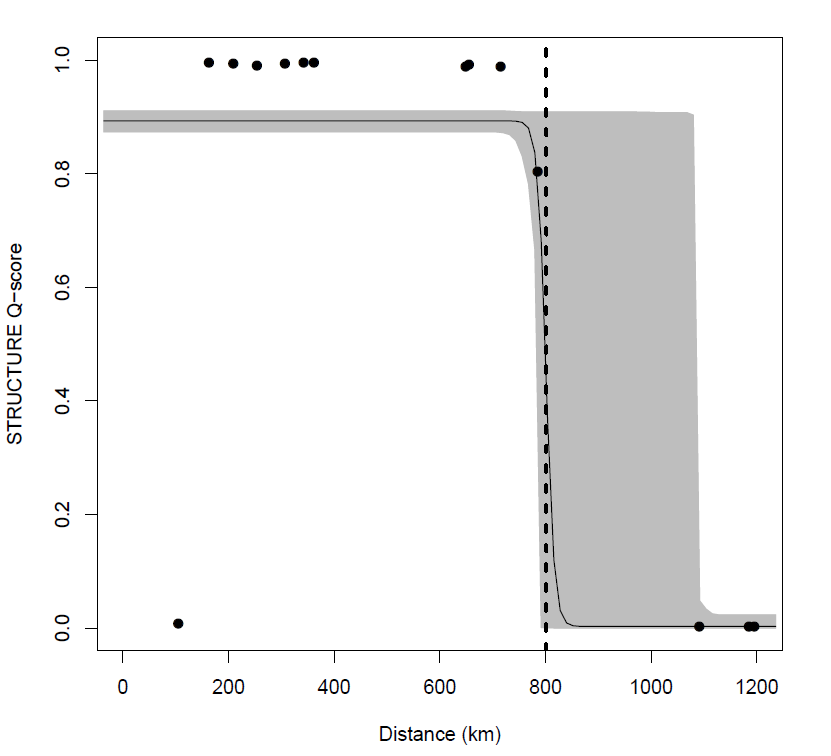


B)
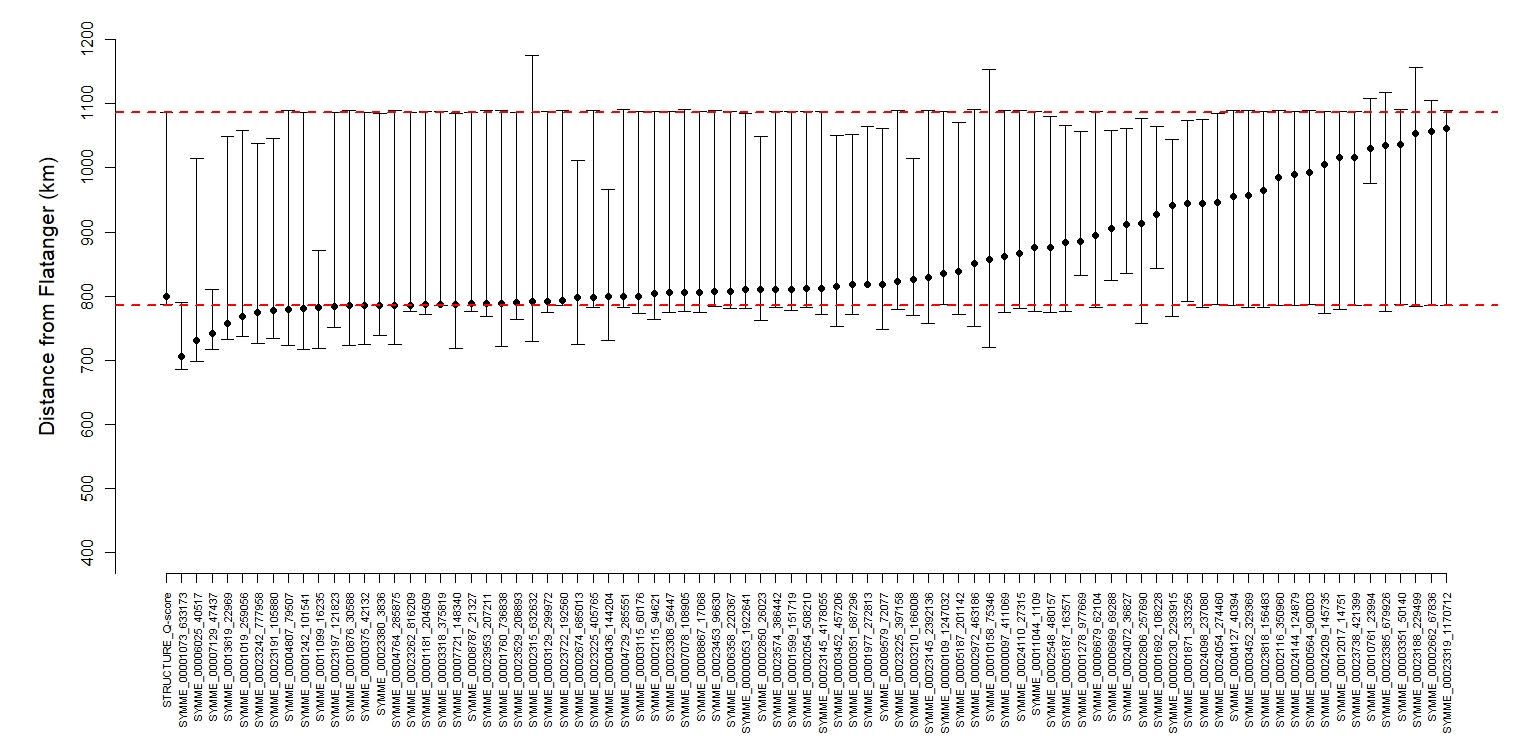


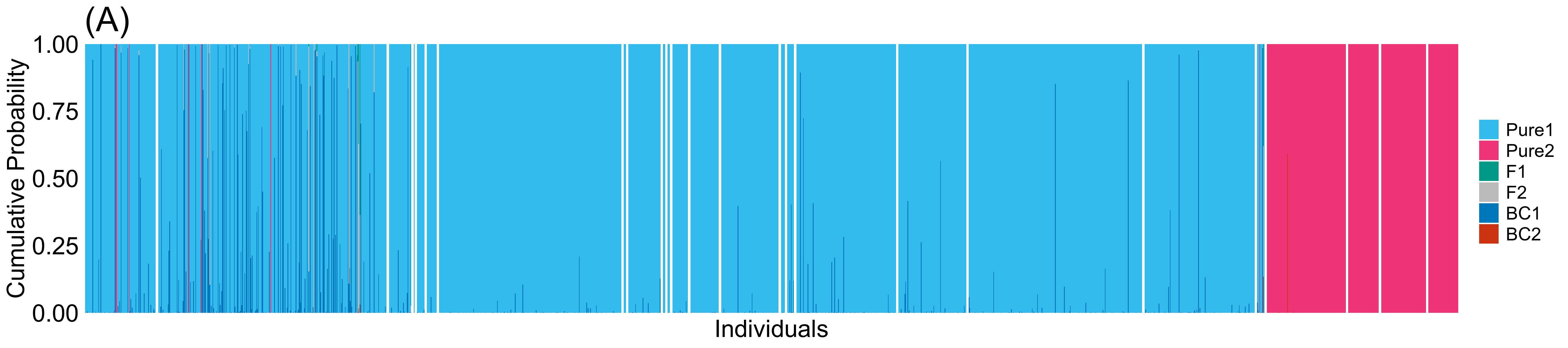
Figure S10. Hybrid analysis of (A) all 1766 individuals using 84 SNPs and (B) zoomed in on individuals sampled in Flatanger 2016, 2017 and 2018. Each line represents one individual and its probability to belong to one of the six genotype classes: pure 1, pure 2, F1 hybrid, F2 hybrid, or backcrosses between F1 and pure 1 or pure 2. Population samples are separated by a whit line and are order from North to South following the coast line. Out of the 432 individuals from Flatanger, we discovered six individuals with clear south-eastern genotypes, two first generation hybrids, and 70 potential second-generation hybrids.


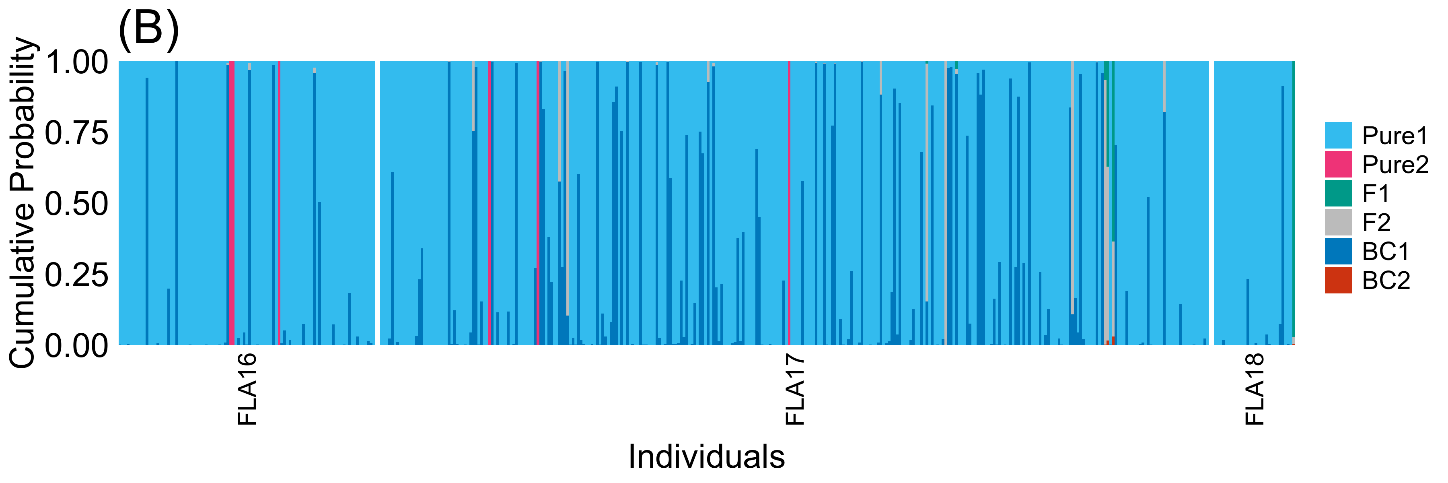


Figure S11. Age distribution of hybrid and pure corkwing sampled in Flatanger 2016 and 2017.


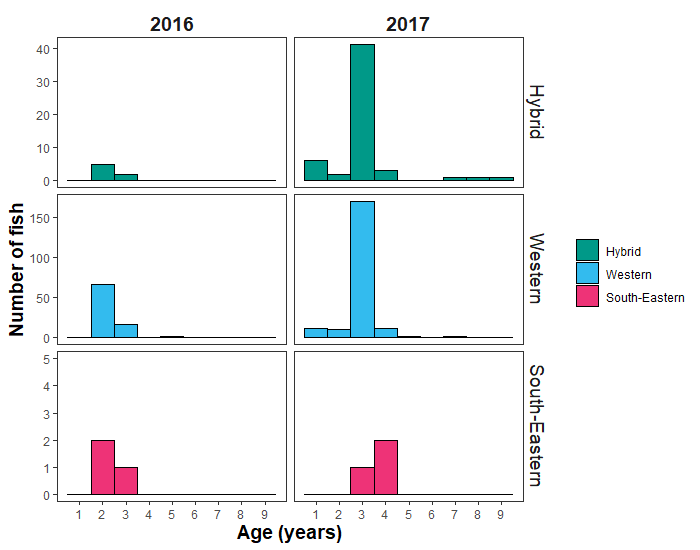


Figure S12. Cleaner fish use in Norway. Figure a) shows the total amount of fish used from 1998 to 2019 (black line and scale on left) and the market value in Norwegian crowns (red line and scale on right). Both scales are given in millions. Figure b) shows the distribution of fish used in 2015-2019. Source: Norwegian Directorate of Fisheries.

a)


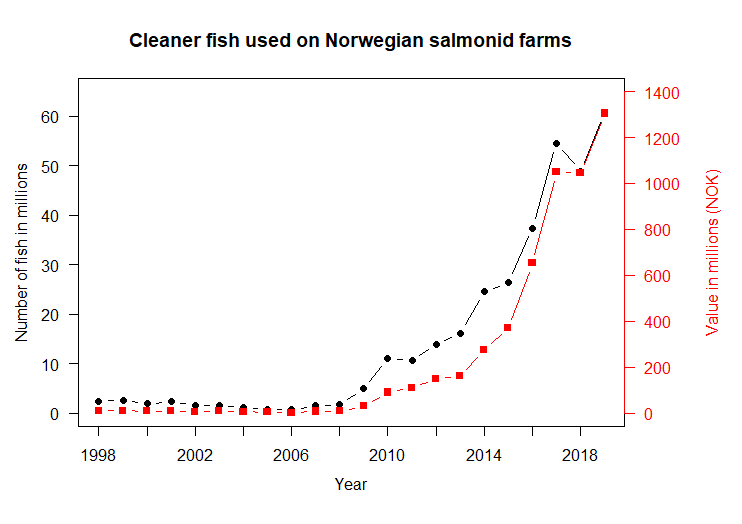


b)


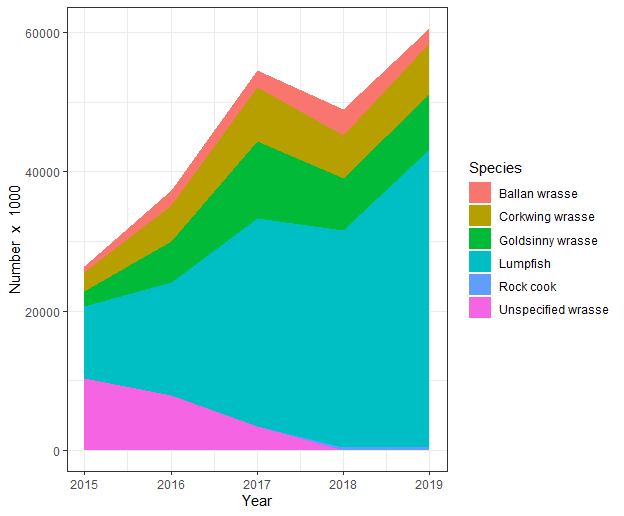


Figure S13. Map of Norway showing number of (A) caught wrasse, (B) wrasse deployed, (C) destination of imported wrasses from Sweden, (D) caught corkwing, (E) corkwing deployed, (F) destination of imported corkwing from Sweden, in 2017 and 2018 (total) for each county. (G) Map of counties.


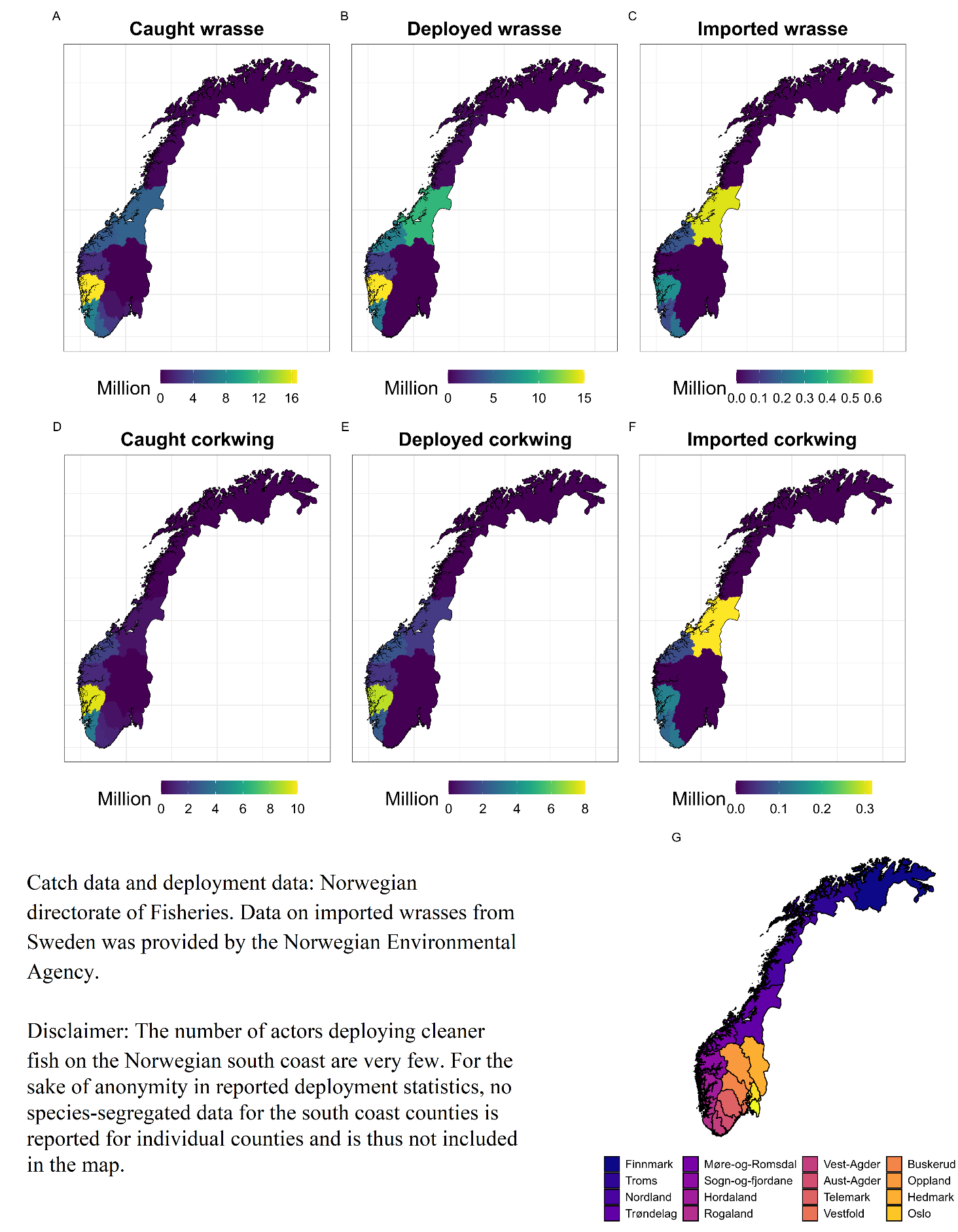

Supplement: Supplementary file 1 — Supplementary Material [file EVA-14-1572-s001.docx]
